# Mixed interactions among life history stages of two harvested related species

**DOI:** 10.1002/ece3.8530

**Published:** 2022-03-07

**Authors:** Edwige Bellier

**Affiliations:** ^1^ Department of Arctic and Marine Biology The Arctic University of Norway Tromsø Norway

**Keywords:** age/stage‐structure, climate variability, compensatory dynamics, harvest, hindcasting, interspecific interactions, marine fish populations, sustainable

## Abstract

Climate change and harvesting can affect the ecosystems' functioning by altering the population dynamics and interactions among species. Knowing how species interact is essential for better understanding potentially unintended consequences of harvest on multiple species in ecosystems. I analyzed how stage‐specific interactions between two harvested competitors, the haddock (*Melanogrammus aeglefinus*) and Atlantic cod (*Gadus morhua*), living in the Barents Sea affect the outcome of changes in the harvest of the two species. Using state‐space models that account for observation errors and stochasticity in the population dynamics, I run different harvesting scenarios and track population‐level responses of both species. The increasing temperature elevated the number of larvae of haddock but did not significantly influence the older age‐classes. The nature of the interactions between both species shifted from predator‐prey to competition around age‐2 to ‐3. Increased cod fishing mortality, which led to decreasing abundance of cod, was associated with an increasing overall abundance of haddock, which suggests compensatory dynamics of both species. From a stage‐specific approach, I show that a change in the abundance in one species may propagate to other species, threatening the exploited species' recovery. Thus, this study demonstrates that considering interactions among life history stages of harvested species is essential to enhance species' co‐existence in harvested ecosystems. The approach developed in this study steps forward the analyses of effects of harvest and climate in multi‐species systems by considering the comprehension of complex ecological processes to facilitate the sustainable use of natural resources.

## Graphical abstract

I analyzed how stage‐specific interactions between two harvested competitors, the haddock (*Melanogrammus aeglefinus*) and Atlantic cod (*Gadus morhua*), living in the Barents Sea affect the outcome of changes in the harvest of the two species. From a stage‐specific approach, I show that a change in the abundance in one species may propagate to other species, threatening the exploited species' recovery.

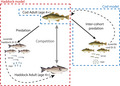

## 1 INTRODUCTION

Species interactions define the functioning of food webs and ecosystems. Indeed, variations of the abundance of species in dynamical systems such as food webs are influenced by trophic interactions within and between species (Estes et al., 2011; Yodzis, 1981). The complexity of food web functioning makes it difficult to demonstrate and quantify interactions (Naeem et al., 1994). Considering the different life history stages of the interacting species is essential to facilitate our ability to quantify interactions among species (Osenberg et al., 1992; Werner & Gilliam, 1984). Stage structure can give rise to a shift in intraspecific and interspecific interactions and affect population and eco‐evolutionary dynamics (Miller & Rudolf, 2011; Stouffer et al., 2012). Harvest and climate are two abiotic factors that could differently affect the stages of the population and disturb their role in the ecological system (May et al., 1978). Many organisms present changes in their developmental history within their lifetime (i.e., ontogeny). Due to ontogenetic niche shift, two individuals can interact either competitively if they are within a similar stage or as predator and prey if the size of one individual corresponds to the preferred prey size range of the other (Polis et al., 1989; Werner & Gilliam, 1984). Therefore, their ecological role is often determined by an individual's size or stage rather than its species identity. This can complicate the characterization of the interactions between two populations presenting ontogenetic changes as “competitive” or “predator‐prey.” Hence, the species level of interaction type is likely to be a complex mixture that depends on the population's stage structure (Holt, 1977; Polis et al., 1989). Individuals typically feed on prey from successively higher trophic levels throughout ontogeny and undergo shifts in the trophic niche (Sibly et al., 2015; Woodward & Hildrew, 2002). The size‐ or stage‐specific nature of these interactions is critically important in shaping species life histories. Changes in such stage‐specific interactions can result in a series of events that can alter the structure in abundance of a community of species (Holt, 1977). Mixed competition‐predation systems induced by size representing complex specific interactions between different stages are widespread in aquatic systems. Therefore, it is crucial to analyze the nature of these interactions and their consequences on population dynamics because they are essential to maintain the proper functioning of interactions in ecosystems (De Roos et al., 2008; Persson & De Roos, 2012). Such systems have even been postulated to explain the lack of recovery of fish stocks (Walters & Kitchell, 2001).

Climate change and harvesting can affect the functioning of different ecosystems (i.e., terrestrial and aquatic) by affecting the population dynamics and interactions among species. Environmental variations can vary the strength of interactions between species through time (Stenseth et al., 2002). Moreover, the demographic structure of one species can be shifted by climate change or harvest. In that case, other species of the ecosystem can be affected, as the type and strength of interactions will be altered. Life history changes of a harvested population affect its population growth and affect the population with which a harvested population interacts, which can threaten the recovery of the exploited species. A change in the abundance or biomass in one species may propagate to other species (Frank et al., 2007; Volterra, 1926). The exploitation of food webs most of the time left out ecological processes (i.e., predation, competition, effects of environmental variations on inter‐ and intra‐specific population processes), resulting in the overexploitation of natural populations and the degradation of their ecosystems (Link, 2002; Pikitch et al., 2004). Overexploitation of fisheries resources can also happen due to insufficient management infrastructure or unbalanced food provision and profits (Costello et al., 2016). Furthermore, coordinated management with ecosystems' ecological properties is crucially needed for strengthening conservation and economic outcomes in wildlife and natural resource management (White et al., 2012). The exploitation of ecosystems based on single‐species management can have critical consequences for the whole ecosystem by causing trophic cascades that disturb the overall functioning of the ecosystems as well as in terrestrial ecosystems (Schmitz et al., 2000) than in aquatic (Carpenter et al., 2001) and marine ecosystems (Frank et al., 2005). As a result, the *Ecosystem*‐*based Approach to Fisheries management* (EAF) has been advocated for several decades (Botsford et al., 1997; May et al., 1979). Nevertheless, EAF is rarely implemented in tactical management (Skern‐Mauritzen et al., 2016).

**FIGURE 1:**
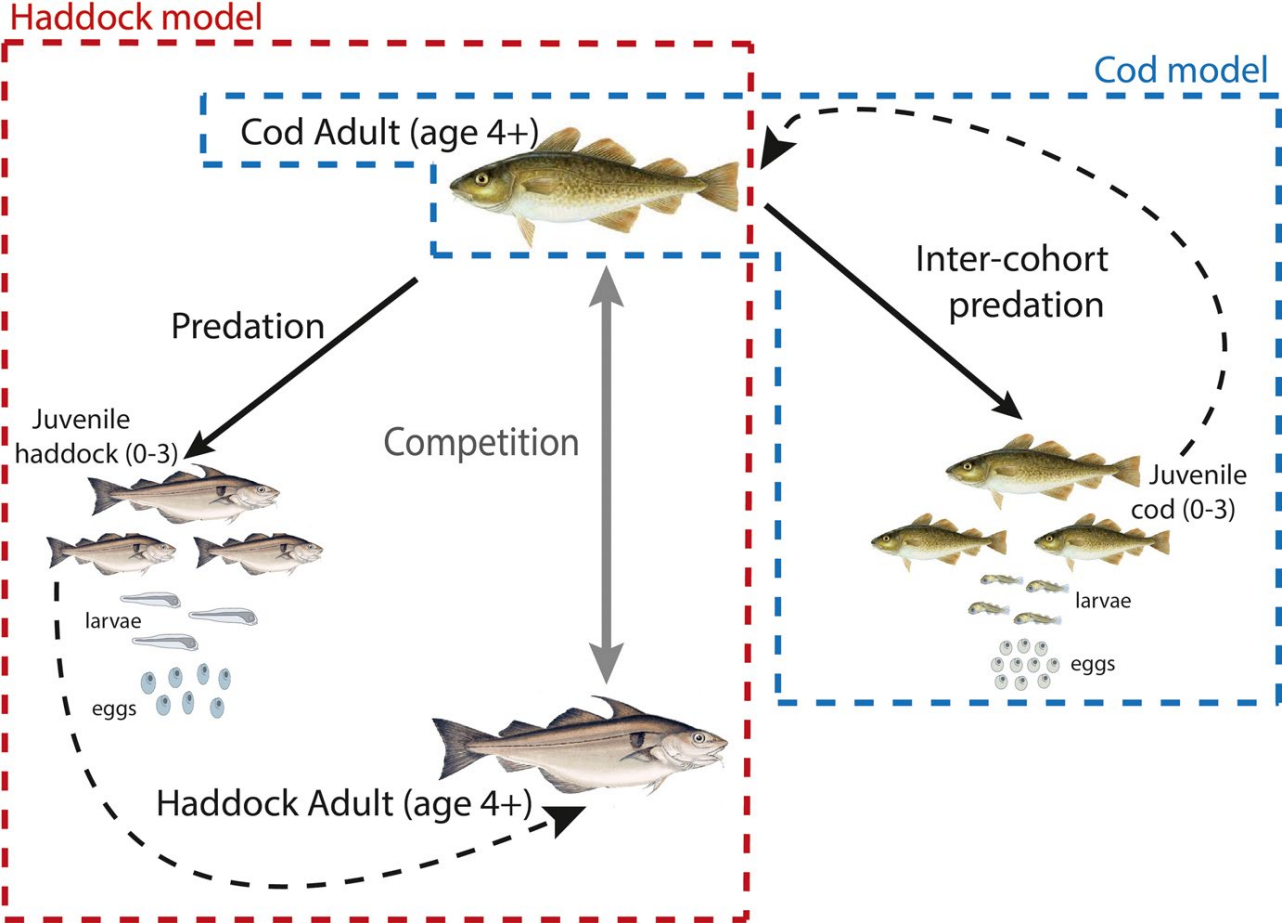
Hypotheses about the inter‐specific interactions among life‐history stages of cod and haddock. Cod is a predator of young haddock stages and also of its own species young stages (black arrows). At adult stages, both species are competitors (grey arrow). Haddock survivors of the juvenile stages enter the adult population (dotted black arrow). Cod survivors of the juvenile stages join the adult population (dotted black arrow). The dotted red lines frame the interactions included in the haddock life cycle model. The light blue dotted lines frame the interactions included in the cod life cycle model. Adult cod is the element common to both haddock and cod models

Here, I present a semi‐integrated two‐species stage‐specific state‐space model that can capture the population dynamics of both species, based on estimated species interaction parameters. I use the haddock (*Melanogrammus aeglefinus*) as a study model to analyze stage‐dependent interactions with its predator and competitor species, the Atlantic cod (*Gadus morhua*) (Figure 1). Competition is an interaction that can shape the variation of abundances of marine fish populations. However, competition is challenging to demonstrate and quantify. Competitive species must show opposite population trajectories, high spatio‐temporal overlap, high dietary similarity and some indications of resource limitations (Link & Auster, 2013).

Haddock and cod are possible competitors at the larval stage, as they have an overlapping diet. Adults have more specific niches (e.g., vertical segregation) though their spatio‐temporal distribution is overlapping. However, their interactions have been little studied (Johannesen et al., 2012). As an adult, cod is a predator of haddock juveniles in the Barents Sea as the weight proportion of haddock in the cod diet is about 6%–14% (ICES, 2008; Link et al., 2009). Predation on haddock juveniles by cod might affect the survival of young haddock; nonetheless, the absolute effect has not been estimated (ICES, 2008; Olsen et al., 2010). The predation of haddock on cod is minimal as haddock eat 0.4% of cod (ICES, 2008). Therefore, the effect of haddock on juveniles of cod seems much less important than the effect of cod on juvenile mortality of haddock (i.e., cod eat 6%–14% of haddock) and the effect of cod intraspecific cohort interaction (i.e., cannibalism) (ICES, 2008, 2013). The cod is also a predator of the capelin (*Mallotus villosus*) and herring (*Clupea harengus*) (Johannesen et al., 2012). Other species such as long rough dab (*Hippoglossoides platessoides*), saithe (*Pollachius virens*), and Greenland halibut (*Reinhardtius hippoglossoides*) are predators of the haddock (ICES, 2008, 2013). Hence, mixed predation‐competition interactions should be considered in the way fisheries are monitored (Link & Auster, 2013; Persson & De Roos, 2012). In this study, I analyze the interactions between haddock and cod as a two‐species study system since the shift of the interaction type through age is identified (Figure 1).

First, I quantify the variation of the strength of interactions between the two species by developing a state‐space model (Aeberhard et al., 2018; Cadigan & Marshall, 2016; Millar & Meyer, 2000; Ohlberger et al., 2014), which includes interactions among life history stages of two harvested interacting species that accounts for observation errors. The model also allows analyzing the sensitivity of the two species to variation in the strength of the species interactions due to variation in harvest and climate. Second, I evaluate the effect of various harvest intensities on one of the competitors (i.e., cod) on the fluctuations in abundances of the other competitor (i.e., haddock) by using a hindcasting approach that allows predicting population's trajectories over a past period. For instance, hindcast has been used to analyze the impact of historical warming on fisheries productivity (Free et al., 2019), with multistate models to compare the habitats of endangered species (Zweig & Kitchens, 2014). Furthermore, the hindcasting enables to simulate perturbations over the past period during which population states and environmental conditions are known (Ohlberger & Langangen, 2015). This approach relies on estimated parameter distributions from a dynamic population model. Thus, it avoids the shortcoming of forecasting, which can lead to an inaccurate accounting of uncertainty and confidence in model projections (Brander et al., 2013). In this study, I use hindcasting to quantify the range of potential impact of different harvest intensities of a predator species on prey abundances changes at different life stages through direct comparison of simulated and historical time series.

## 2 MATERIALS AND METHODS

### 2.1 Study system

The study system comprises two Gadidae species, the Northeast Arctic (NEA) haddock and the NEA cod, which are batch‐spawners with high fecundity, feeding on smaller fishes and crustaceans. Both species live in the same area, the Barents Sea, a high‐latitude shallow sea located to the north of Norway and Russia (Bakketeig et al., 2016; Olsen et al., 2010). The Barents Sea is huge (1.6 million square kms), distributed across several degrees of latitude, and presents a variety of unique thermal habitats. These two species live in sympatry in the Barents Sea and co‐occur almost everywhere in the area (Figure S1). The productive Barents Sea ecosystem has been affected by human activity for several hundred years, mainly by fishing and hunting (Shevelev et al., 2011), and more recently by climate warming. The warming trend in the Barents Sea is on average about 1°C over the 2000s (Lind et al., 2018). The sea surface temperature (SST) is higher in regions under the influence of the warm Atlantic Current (South West, Bear Island Trend, Thor Iversen Bank, and South East); the lowest SST is observed in the northern area of the Barents Sea, which is influenced by the Arctic waters (Dalpadado et al., 2020). Moreover, the winter sea‐ice extent has decreased by approximately 50% over the last 20 years (Skagseth et al., 2020). Therefore, the Barents Sea is dramatically affected by changes in temperature and sea ice cover. These changes might alter the Arctic marine ecosystems and modify the biological diversity toward more boreal and fewer Arctic species (Dalpadado et al., 2020). The variability in the ecosystem's productivity may substantially impact the large and economically crucial fish stocks in the area. Haddock is most abundant along the coast over the continental shelf, abundant along the Norwegian coast, and the Barents Sea (Olsen et al., 2010). Northeast Arctic haddock spawn pelagic eggs from March to June, with a maximum in late April and early May. NEA Cod spawn along the Norwegian coast from February to April. NEA Cod eggs drift with the Norwegian Coastal Current along the Norwegian coast toward the northeast and hatch within a month. Pelagic juveniles (0‐group) get to the Barents Sea in late summer and remain in this area until they reach maturity (6–8 years old). The feeding area of both species and their prey overlap mainly early in life (Kane, 1984; Olsen et al., 2010). Spatio‐temporal overlap between both species, development stages, size, and stock state may influence species interactions' strength and type (Langangen, Stige, Yaragina, Vikebø, et al., 2014). Cod can feed on haddock's juveniles in large quantities in some years; the consumption was especially high in 1986, 1992, 1995, and from 2005 to 2012 (ICES, 2008, 2013).

### 2.2 Age structured life cycle population model

In ecology, state‐space models are widely used to analyze ecological times series and population dynamics (Clark, 2007; Dennis & Taper, 1994; Knape, 2008). This kind of model enables separate ecological process variation (i.e., stochastic process) and observation errors (de Valpine & Hastings, 2002). This study is based on state‐space models that describes individual species life cycle models (Millar & Meyer, 2000; Ohlberger et al., 2014). For a trade‐off in model complexity, I extended and corrected the haddock life cycle model of Patin et al. (2015) by removing the residual variability in fishing mortality that was independent of year and age (i.e., reflected by *τ_W_*), and fixed the age‐specific mortality for haddock older than 4 years. I reduced the model by excluding the third prey species (i.e., capelin) to focus on analyzing of the mixed competition‐predation system (i.e., haddock and cod). Furthermore, I included species interactions that account for all potential predation. In contrast, Patin et al. (2015) considered only a part of the potential predation by not including the older age‐classes and the non‐spawning individuals.

#### 2.2.1 Age‐specific abundance process model for haddock

Here, the process model describes the age‐structure dynamics of one species (i.e., haddock) starting from the production of recruits in the population and include only the predation by the other species (i.e., included as the biomass of the mature population) on the juvenile's stage (see Tables 1 and 2 for parameters definition). I assume that the number of juveniles $Nh_{0,y}$ born each year, *y*, depends on temperature. Indeed, some studies show that warming temperature contributes to higher survival of early life stages of gadoids (Ottersen & Loeng, 2000; Stige et al., 2010). Thus, the number of juveniles can be obtained from a stochastic stock‐recruitment relationship estimated from the mean number of juveniles (Beverton & Holt, 1957; Ricker, 1954). Spawning stock biomass *Sh_y_* of haddock (*h*) was estimated as a function age‐specific abundances *Nh_a_* from age *a* to *A*, weight‐at‐age *Wh_a_*_,_ *_y_* and the probability of being mature *Ph_a_*_,_ *_y_*,

(1) $$Sh_{y}=\sum_{a=1}^{a=A}Nh_{a,y}Ph_{a,y}Wh_{a,y},$$

**TABLE 1:** Description and definition of each variable of the haddock model (Eq. 1 to Eq. 12)

Variable	Definition	Equations	Type
*h*	Haddock	1	index
*S_y_*	Spawning stock biomass at year y	1	Estimate
*a*	Age	1	index
*N_a_*_,_ *_y_*	Age‐specific abundance at age *a* and year *y*	1	Estimate
*P_a_*_,_ *_y_*	Probability of being mature at age *a* and year *y*	1	Data
*W_a_*_,_ *_y_*	Weight‐at‐age at age *a* and year *y*	1	Data
*N_0_*_,_ *_y_*	Number of juveniles at year *y*	2	Estimate
$\varphi_{y}$	Mean fecundity at year *y*	2	Estimate
*m_e_*	Mortality between egg to 0‐group	2	Estimate
*δ_T_*	Correction factor for the fecundity *φ_y_*	3	Estimate
$\sigma_{\varphi }^{2}$	Process error variance of the estimate of the number of juveniles *N_0_*_,_ *_y_*	2	Estimate
$T_{\varphi }$	Temperature dependent factor	3	Estimate
*T_y_*	Temperature at year *y*	3	Data
$\bar{T}$	Mean temperature from December to February	3	Estimate
*Z_a_*_,_ *_y_*	Mortality rates at age *a* and year *y*	5	Estimate
*M_a_*_,_ *_y_*	Natural mortality at age *a* and year *y*	5	Estimate
*F_a_*_,_ *_y_*	Fishing mortality	5	Estimate
*m_a_*	Age‐specific mortality *a*	6	Estimate
*δ_a_*	Constant allowing unbiased estimates	6	Estimate
*g_a_*	Age‐specific predation term	6	Estimate
*Gy*	Predator mature population biomass estimates	6	Data
*D_a_*	Age‐specific density‐dependent term	6	Estimate

**TABLE 2:** Following of the description and definition of each variable of the haddock model (Eq. 1 to Eq. 12)

Variable	Definition	Equations	Type
*f_a_*	Age‐specific selectivity	8	Estimate
*e_y_*	Log effort of harvest at year *y*	8	Estimate
$\sigma_{F}^{2}$	Variance of the log effort of harvest	8	Estimate
$\sigma_{w}^{2}$	Variance of the random term	8	Estimate
*C_a_*_,_ *_y_*	Catch for a given age *a* and year *y*	10	Estimate
*L_a_*_,_ *_y_*	Reported landings	11	Estimate
$\varepsilon_{L_{a,y}}$	Gaussian errors term associated to estimated catches	11	Estimate
$\sigma_{L_{a}}$	Variance associated to the Gaussian errors of the estimated catches	11	Estimate
*I_a_*_,_ *_y_*	Abundances indices at age *a* and year *y*	12	Estimate
*q_a_*	Proportionality constants reflecting age‐specific catchability estimates	12	Estimate
$\varepsilon_{I_{a,y}}$	Gaussian error term associated to the abundance's indices	12	Estimate
$\sigma_{I_{a}}$	Age specific observation errors variance	12	Estimate
$\xi_{y}$	Random year effect that accounts for correlated observation errors within years	12	Estimate
$\sigma_{\xi }$	Variance of the random effect of observation errors within years	12	Estimate

The mean number of juveniles was estimated using fecundity estimates in number of eggs per gram and mortality from eggs to 0‐group. Based on Ohlberger et al. (2014), the number of juveniles of haddock *Nh_0_*_,_ *_y_* depends on temperature and is expressed as an exponential relationship such as,

(2) $$\text{ln}\left(Nh_{0,y}\right)∼N\left[\text{ln}\left[Sh_{y}\varphi_{y}\exp\left(‐m_{e}\right)\right]‐\frac{\sigma_{\varphi }^{2}}{2},\sigma_{\varphi }^{2}\right]$$

where $\sigma_{\varphi }^{2}$ is the process error variance, and *m_e_* is the mortality during the period from egg to 0‐group survey; then the mean fecundity *φ* is expressed such as,

(3) $$\varphi_{y}=\varphi \exp\left[\delta_{T}+T_{\varphi }\left(T_{y}‐\bar{T}\right)\right]$$

where *T_φ_* is the temperature‐dependent factor, $\bar{T}$ is the mean temperature from December to February, and $\delta_{T}$ a correction factor to get an unbiased mean fecundity,

(4) $$\delta_{T}=\frac{Y}{\sum_{y}\exp\left[T_{\varphi }\left(T_{y}‐\bar{T}\right)\right]}$$

For the age‐classes >0, the number of fish *Nh* at age *a* and in the year *y* is expressed as a function of the population size of the previous year for a given age and *Zh_a_*_,_ *_y_* as its mortality rates.

From Aanes et al. (2007), the mortality is decomposed as a natural mortality $Mh_{a,y}$ and a fishing mortality $Fh_{a,y}$ (i.e., harvest mortality) terms,

(5) $$Zh_{a,y}=Mh_{a,y}+Fh_{a,y}.$$

For fish younger than four‐years‐old (*a* < 4), the natural mortality is decomposed in a fixed mortality, an exponential function of a density‐dependent term expressed as a Beverton‐Holt relationship and a predation term such as,

(6) $$Mh_{a,y}=m_{a}\exp\left(\delta_{a}+g_{a}G_{y}\right)+\text{ln}\left(1+Dh_{a}Nh_{a,y}\right),$$

where $m_{a}$ is an age‐specific mortality, $g_{a}$ is the age‐specific cod predation term, and $G_{y}$ is an estimate of the biomass of the mature population (i.e., cod population). Predation was assumed to be negligible for ages 4+ because the main predator generally preys on smaller individuals and mortality due to fishing is much higher than by predation. *Dh*_a_ is an age‐specific density‐dependent term and $\delta_{a}$ is a constant that allows for an unbiased estimate of the mean mortality, such as,

(7) $$\delta_{a}=\frac{Y}{\sum_{y}\exp\left(g_{a}G_{y}\right)},$$

where *Y* is the number of years. Density‐dependence in survival was assumed to be negligible for age‐4 and older fish.

The fishing mortality *Fh_a_*_,_ *_y_* is decomposed as in Aanes et al. (2007) into (i) an age‐specific selectivity term *f_a_*, for age‐3 and older individuals, (ii) a year‐specific term *e_y_*, which is a random noise with variance $\sigma_{F}^{2}$ to be estimated,

(8) $$\text{ln}\left(Fh_{a,y}\right)∼N\left(\text{ln}\left(f_{a}\right)+e_{y}\right)$$

(9) $$e_{y+1}∼Nh\left(e_{y},\sigma_{F}^{2}\right).$$

Finally, the latent catches correspond to the fraction of the total removal of individuals from the population due to harvest. From the Baranov catch equation (Quinn & Derison, 1999), the catch for a given age and year *Ch_a_*_,_ *_y_* is related to age‐specific abundances through,

(10) $$Ch_{a,y}=\frac{Fh_{a,y}}{Fh_{a,y}+Mh_{4+}}Nh_{a,y}\left(1‐e^{‐\left(Fh_{a,y}+Mh_{4+}\right)}\right).$$

#### 2.2.2 Observation model for haddock

The haddock observation model enables to connect the latent abundance to the observations. Based on Ohlberger et al. (2014), I used lognormal errors (Millar & Meyer, 2000) to link the latent variables (i.e., *abundances* and *catches*) to the survey *indices* and reported *landings*. I assumed that the errors in reported landings ($Lh_{a,y}$) were log‐normally distributed and independent between ages and years such as,

(11) $$Lh_{a,y}=Ch_{a,y}e^{\varepsilon_{Lh_{a,y}}‐0.5\sigma_{Lh_{a}}^{2}},$$

where the Gaussian error term is $\varepsilon_{Lh_{a,y}}$ with mean zero and variance $\sigma_{L_{a}},$ the mean of the catch error $0.5\sigma_{L_{a}}^{2}$ is adjusted to one any value of $\sigma_{Lh_{a}}$. The correction term was not explicitly included in the abundance indices because it only acts on the values of the age‐specific catchability estimates. The indices of age‐specific abundance issued from the scientific surveys were assumed to be related with observation error due to the sampling. Abundance indices (age‐classes 1–9) were expressed as,

(12) $$Ih_{a,y}=q_{a}Nh_{a,y}e^{\varepsilon_{I_{a,y}}\xi_{y}},$$

where Gaussian term is $\varepsilon_{I_{a,y}}$ and has a zero mean and variance $\sigma_{I_{a}}$; the random year effect, $\xi_{y}∼Nh\left(0,\sigma_{\xi }\right)$, accounts for correlated observation error within years. The proportionality constant *q_a_* describes the age‐specific catchability at the time of the survey.

#### 2.2.3 Age‐specific abundance process model for cod

The cod process model is a dynamic age‐structured population model which accounts for environmental stochasticity on age‐specific survival through lognormal process noise (Ohlberger et al., 2014). The estimation of the first stage of the life cycle (i.e., the number of eggs *NC_e_* of cod *C*) at a given year *y*, is based on the estimation of the stock spawning biomass *SSB* at a given year *y* such as,

(13) $$SSB_{y}=\sum_{a=1}^{a=A}NC_{a,y}PC_{a,y}WC_{a,y},$$

where *NC_a_*_,_ *_y_* is the specific abundance of cod *C* at age *a*, *PC_a_*_,_ *_y_* is the probability of being mature, and *WC_a_*_,_ *_y_* is the weight at a given age, the maximum age is *A*. The number of eggs is defined such as,

(14) $$NC_{e,y}=SSB_{y}\tau e^{\varepsilon_{\mathrm{sp}}},$$

where the mean fecundity (eggs kg^−1^) is *τ*, and the stochasticity in the spawning process *ε_sp_* is a Gaussian error term with mean zero and variance *σ*_sp_ (Marshall et al., 2006). The number of larvae *NC_l_* is estimated from the egg abundance and mortality (*M_e_*, day^−1^) between egg and larvae survey (*t_e_*, days) such as,

(15) $$NC_{l,y}=NC_{e,y}e^{‐t_{e}M_{e}}.$$

Temperature effects are included on larval survival because warmer temperature is associated with higher survival of early life stages of cod, especially larvae (Ottersen & Loeng, 2000). Thus, the number of 0‐group of cod *NC_z_* is estimated from the number of larvae as,

(16) $$NC_{z,y}=N_{Cl,y}e^{‐t_{l}M_{l}+\psi_{l}T_{l}},$$

*M_l_* is the cod mortality between the larvae and 0‐group survey (*t_l_*, days), and *ϕ_l_ T_l_* is a temperature effect estimated from the thermal conditions through the larval stage (June–August). The number of age 2–4 of cod is estimated from the preceding age‐class and year such as,

(17) $$NC_{a,y}=NC_{a‐1,y‐1}\frac{1}{1+\beta_{a}NC_{a‐1,y‐1}}e^{‐M_{j}},\;\text{for}\;2<a<4,$$

where *M_j_* is the juvenile mortality, and $\beta_{a}N_{a‐1,y‐1}$ is an inter‐cohort interaction term representing the increase in mortality with abundance.

Fishery mortality affects cod from age‐4. Therefore, the number of age 5–9 of cod is estimated from the abundance of the preceding age‐class and year,

(18) $$NC_{a,y}=NC_{a‐1,y‐1}e^{‐\left(F_{a‐1,y‐1}+M_{4+}\right)},\;\text{for}\;5<a<9$$

where $M_{4+}$ is the natural mortality and $F_{a‐1,y‐1}$ is the fishing mortality on that age‐class the previous year. The fishing mortality is expressed such as,

(19) $$FC_{a,y}=e^{f_{a}+f_{y}},$$

where *f_a_* describes the mean relative proportion of fish caught by the fishery for a given age and *f_y_* represents the fishing effort over the years. The fishing effort can change over time. Thus, it is expressed from a random walk defined by a normal distribution $\varepsilon_{f_{y}}∼N\left(0,\sigma_{f_{y}}\right)$ and defined as

(20) $$f_{y}=f_{y‐1}+\varepsilon_{f_{y}}.$$

The catch *CC_a_*_,_ *_y_* for a given age and year is defined as a function of age‐specific abundances,

(21) $$CC_{a,y}=\frac{FC_{a,y}}{FC_{a,y}+M_{4+}}NC_{a,y}\left(1‐e^{‐\left(FC_{a,y}+M_{4+}\right)}\right).$$

The cod catches are defined in the same way as the haddock and are based on the Baranov catch equation (Quinn & Derison, 1999). In addition, the observation model of the cod is similar to the one of haddock (see Eq. 11 and Eq. 12).

### 2.3 Data description and parameter estimation

**FIGURE 2:**
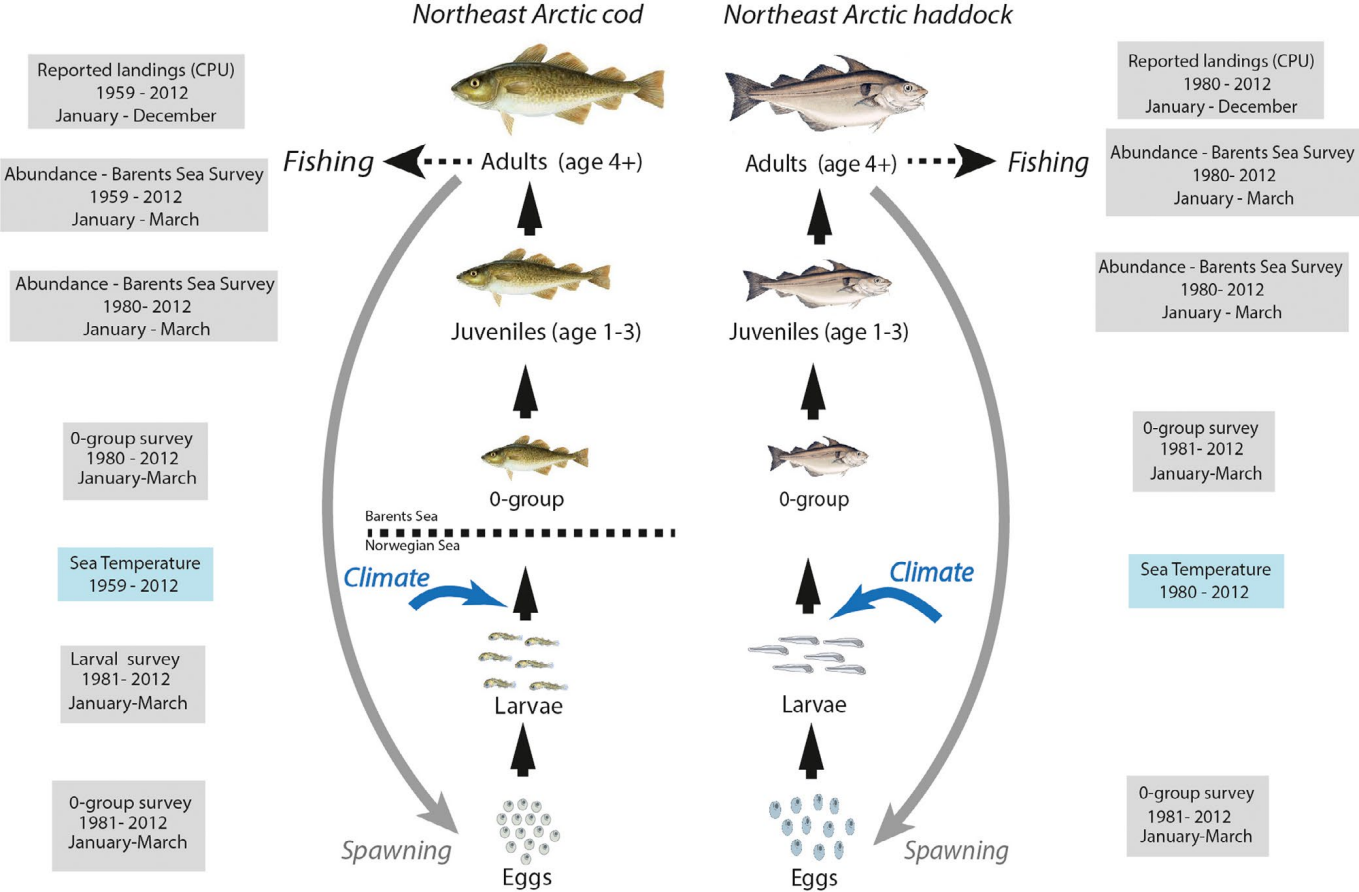
Life cycles of Northeast Arctic cod (left) from Ohlberger et al. (2014) and Northeast Arctic haddock (right), harvest of adults, climate and data structure

The model includes information from scientific surveys and commercial harvest from 1980 until now a day (i.e., from 1980 to 2012, 33 years). Reported landing and abundance estimates for NEA haddock are publicly available in ICES Report (ICES, 2013). A schematic representation of the different data is provided in Figure 2, and more detailed information about the data is given in the Supporting Information. I used a Bayesian framework to estimate the haddock life cycle model's parameters, including prior knowledge about parameter values (see Table S1). The analysis was implemented using JAGS (Plummer, 2003) through R2jags (Su & Yajima, 2012) within R (R Development Core Team, 2019). Markov Chain Monte Carlo (MCMC) runs were composed of three chains with a burn‐in of 250,000 samples and a posterior distribution on 250,000. I used a thinning of 1000 to reduce autocorrelation in chains. I checked the convergence with the Brooks‐Gelman Rubin diagnostic (R‐hat statistics) (Gelman et al., 2004) and by visual inspection of the chains. I considered that the model converged when R < 1.1 for all model parameters and that all chains were well‐mixed.

### 2.4 Analyses of the mixed competition‐predation system

I ran the cod model (Eq. 13 to Eq. 21) to obtain the estimate of cod abundances (from 1959 to 2012). I then estimated the cod biomass from the weights at age provided in the ICES tables (ICES, 2013). I included the estimated cod biomass from age‐3 to ‐12 in the haddock model (Eq. 7) as a covariate (i.e., *G_y_*). I ran the haddock model (Eq. 1 to Eq. 12) and estimated all the haddock model parameters. From the output of the haddock model, I analyzed the effect of predation by cod on the haddock of age‐0 to age‐4. Next, I investigated the effect of the cod inter‐cohort interactions on the abundances of haddock. For this, I analyzed the variation of the estimated log‐numbers of individuals of haddock within each stage from age‐0 to age‐5 as a function of the estimated strength of cod inter‐cohort interaction *β* obtained from the cod model (Eq. 17) by using generalized additive models (GAMs) (Hastie & Tibshirani, 1990; Wood, 2006). This kind of model enables to fit smoothed curves to capture non‐linear relationships between abundances and covariables. Here, to account for uncertainty in the estimates of *β*, the GAMs were weighted by the standards errors of the estimated cod inter‐cohort interaction term. Similarly, I analyzed the effect of temperature on juveniles and adults population size weighted by the standards errors of the estimated juveniles and adults population size.

### 2.5 Design of the hindcasting analyses

**FIGURE 3:**
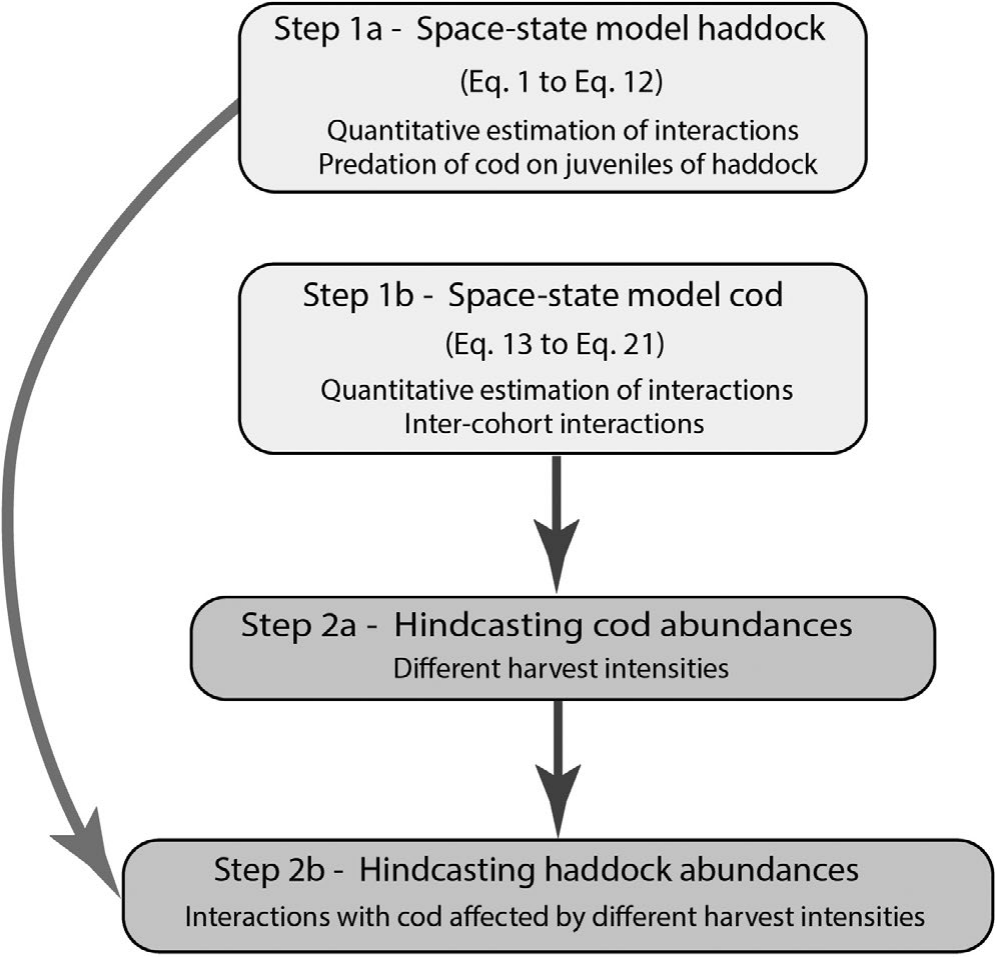
Description of the different steps of the hindcasting framework

**FIGURE 4:**
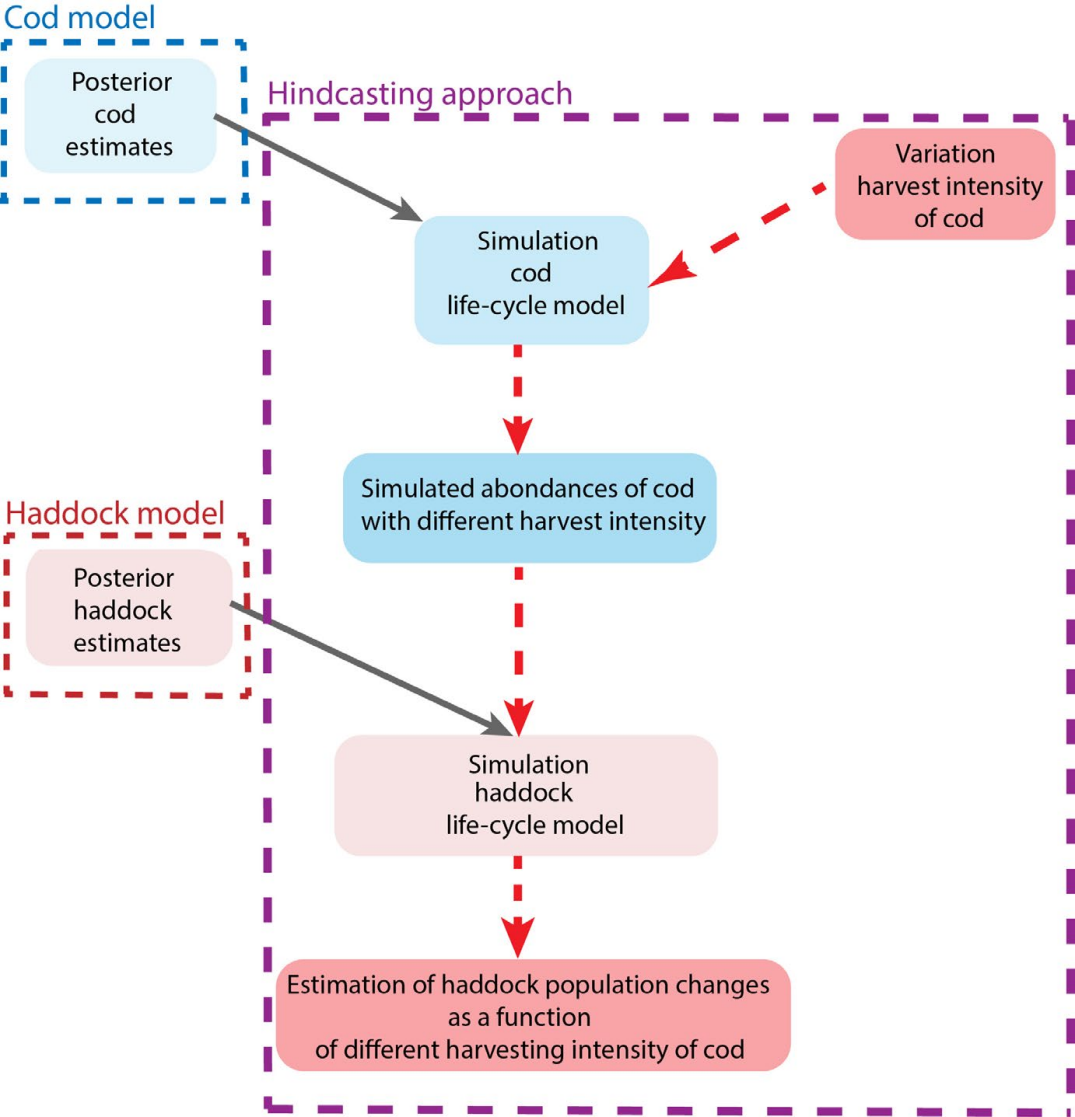
Schematic representation of the hindcasting approach with two interacting species. The simulation of the cod life cycle model is based on Eq. 13 to Eq. 21 and the model of Ohlberger et al. (2014); the simulation of the haddock life cycle model is based on the model described in section “Process age‐specific abundance” and “Observation model” in the main text (see Eq. 1 to Eq. 12). The dotted red arrows represent the propagation of the harvest effects through the life cycle of both species

I integrated the use of two single age‐structured life cycle population models, one for the haddock (Eq. 1 to Eq. 12) and one for the cod (Eq. 13 to Eq. 21) based on Ohlberger et al. (2014), to quantify the range of potential impact of different harvest intensities on cod in terms of population changes of haddock over the past (from 1980 to 2012); see Figures 3 and 4 for a schematic representation of the hindcasting approach. The life cycle model of the population dynamics of cod separates the effects of density‐independent (i.e., temperature) and density‐dependent processes on early life stages of cod (Ohlberger et al., 2014). This age‐structured population model unravels the effects of stochasticity and density regulation on the survival of early life stages (pre‐recruit). The hindcasting approach enables to simulate perturbations over the past period during which population states and environmental conditions are known (Ohlberger & Langangen, 2015). I varied the fishing mortality (i.e., harvest mortality) of the life cycle cod model described in Eq. 13 to Eq. 21 to obtain the estimates of abundances of cod with different harvest intensities (Figure 4). Then, the estimated parameters (posterior distributions) issued from the estimation of the haddock life cycle model (see Eq. 1 to Eq. 12) were used to simulate the consequences of variation of the intensity of the cod's harvest on the variation of abundance of the population of haddock. I ran the hindcast life cycle haddock model specifically with each different abundances of cod affected by given harvest intensities. This enabled to obtain hindcasted predictions of haddock's abundances as a function of abundances of cod affected by varying harvest intensities. Simulations were realized by sampling parameters from the estimated posterior distributions of the age‐specific mortality rates, temperature effect, density dependence terms, fishing mortalities, and process errors (see Tables S3 and S4 for median values and standard deviation of the key model parameters).

## 3 RESULTS

**FIGURE 5:**
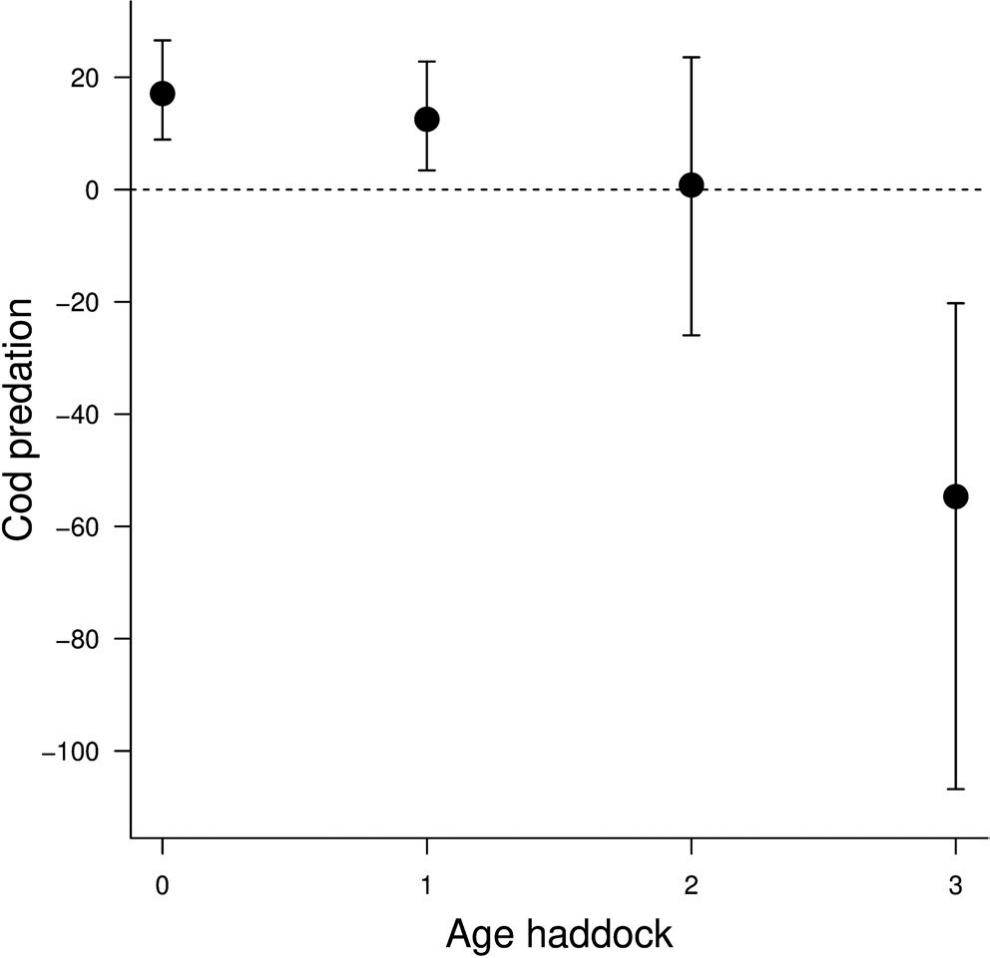
Estimated predation of cod *g_a_* on different life stages of haddock (Eq. 6) from age‐0 to age‐4. The black dots represent the mean estimates of predation of haddock by cod (Eq. 6) multiplied by the overall cod biomass. The vertical lines correspond to the 95% credibility intervals

Predation by cod significantly affects mortality of haddock at age‐0 and age‐1 (Figure 5). The impact of cod predation was not significant at age‐2 and became negative on the mortality of haddock at age‐3 (Figure 5). Mortality of haddock age‐0 and age‐1 increased over the last decades; mortality at age‐0 was the highest and increased since the last 20 years (Figure S3). Mortality at age‐1 was lower than at age‐0 and also increased from 2000 to 2010. The variation of mortality at age‐2 was very low over the study duration. Mortality at age‐3 was the lowest and decreased from 2000 (Figure S3). Mortality at age‐0 was highly density‐dependent (Figure S3) and became less important when the age increased. At age‐3, the density dependence effect on mortality was close to zero (Figure S4).

Overall, the estimated number of individuals for all the age‐classes presented an increasing trend over the study period (Figure S5). Age‐0 is the stage that showed the highest number of individuals overall the age‐classes. Fluctuations in the number of individuals were higher from 1980 to 2000 than after 2000. Noticeably, after 2000, the fluctuations in the number of individuals were lower (Figure S5). Importantly, the number of individuals of the harvested age class estimated by the models aligned with the stock assessment estimations (Figure S5).

**FIGURE 6:**
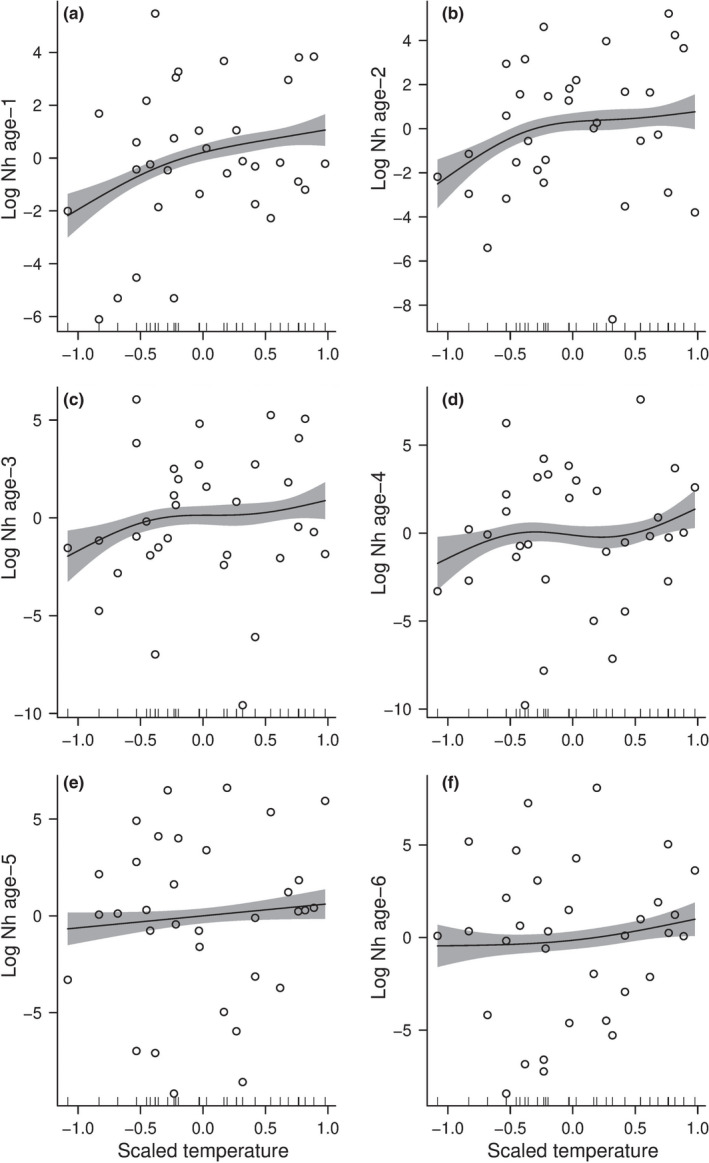
Coefficients of the Generalized Additive Models (GAMs) for the estimated log numbers of individuals of haddock *Nh_a_*_,_ *_y_* as a function of scaled water temperature T. (a–f) Coefficients of the estimated log numbers of individuals from age‐0 to age‐5 as a function of scaled water temperature. The black line corresponds to the value of GAMs coefficient; the grey area represents the 97.5% confidence intervals. The generalized additive model is weighted by the standards errors of the estimated number *Nh_a_*_,_ *_y_* of individuals of haddock

**FIGURE 7:**
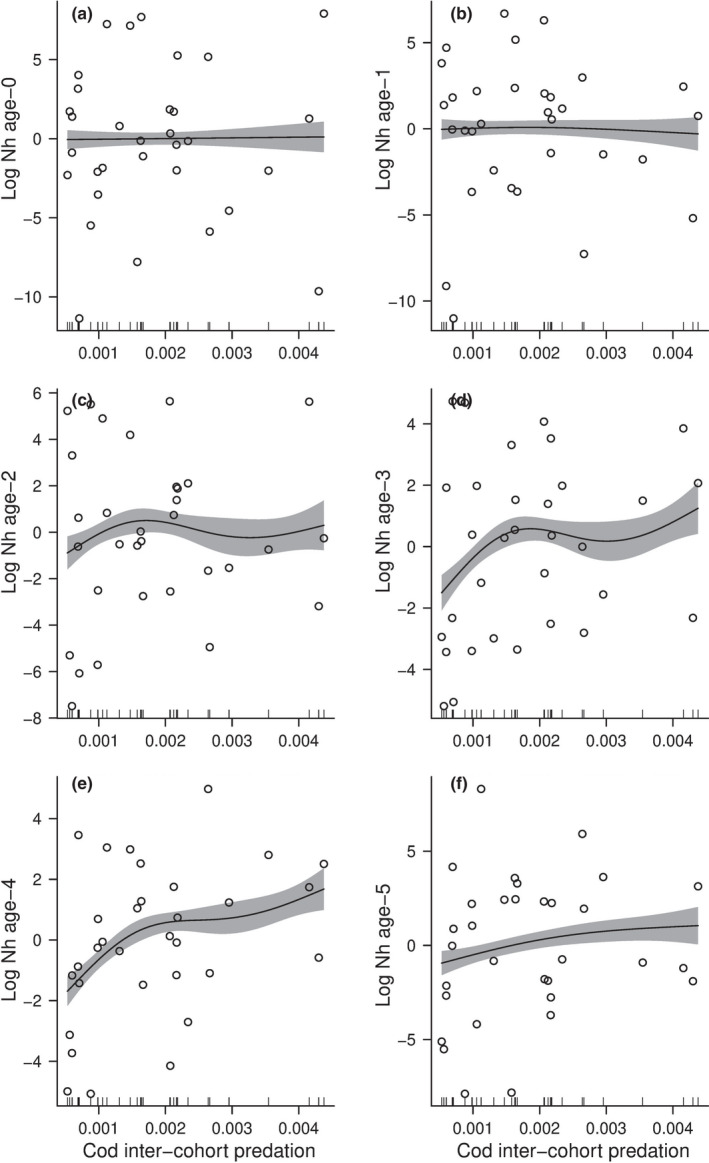
(a–f) Coefficients of the Generalized Additive Models (GAMs) for the estimated log‐numbers of individuals of haddock within each stage from age‐0 to age‐5 as a function of the estimated cod inter‐cohort interaction term γ obtained from the model described in Eq. 13 to Eq. 21 and in Ohlberger et al. (2014). The black line corresponds to the value of GAMs coefficient; the grey area represents the 97.5% confidence intervals. The generalized additive model is weighted by the standard errors of the estimated cod inter‐cohort interaction term

Particularly, the number of haddocks of age‐1 increased as a function of the temperature (% deviance = 64.5, Table 3) (Figure 6a) whereas the temperature had a lower effect on the population which is harvested (% deviance = 28.9, Table 3) (Figure 6e,f). Cod inter‐cohort predation (i.e., cannibalism) did not have so much effect on the number of individuals of haddock at age‐0 (% deviance = 0.21, Table 3) (Figure 7a), age‐1 (% deviance = 3.65, Table 3) (Figure 7b), and age‐2 (% deviance = 7.89, Table 3) (figure 7c). The number of individuals of haddock significantly increased as a function of cod inter‐cohort predation (i.e., cannibalism) at age‐3 (% deviance = 37.1, Table 3) (Figure 7d), age‐4 (% deviance = 70.4, Table 3) (Figure 7e), and age‐5 (*%* deviance = 28.6, Table 3) (Figure 7f).

**TABLE 3:** Single GAM fits for age‐structured haddock as a function of cod inter‐cohort predation and temperature

Parameter	Cod inter‐cohort predation		Temperature	
	% Deviance	GCV score	% Deviance	GCV score
Log Nh 0‐age	0.21	2.83 × 10^−5^	—	—
Log Nh 1‐age	3.65	2.56 × 10^−5^	64.5	8.16
Log Nh 2‐age	7.89	2.25 × 10^−5^	51.1	11.018
Log Nh 3‐age	37.1	1.45 × 10^−5^	32.4	15.16
Log Nh 4‐age	70.4	7.27 × 10^−5^	28.9	18.016
Log Nh 5‐age	28.6	1.57 × 10^−5^	9.59	20.27
Log Nh 6‐age	—	—	18.2	19.63

Note

The percentage of deviance explained by each covariable and Generalized Cross Validation.

**FIGURE 8:**
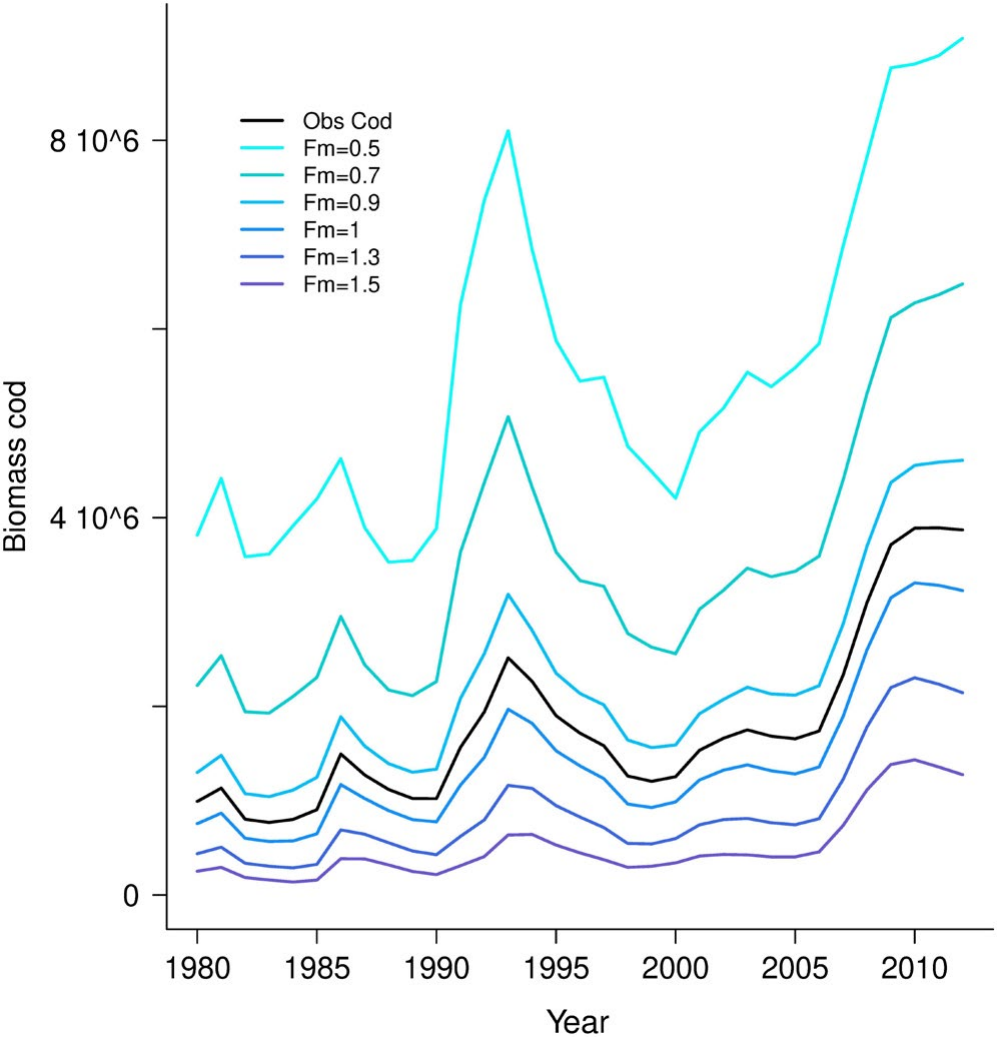
Hindcasted biomass of cod (in metric tons) from the life cycle cod model from 1980 to 2012 as described in Ohlberger and Langangen (2015) with different harvest intensity (*Fm*) ranging from 0.5 to 1.5

**FIGURE 9:**
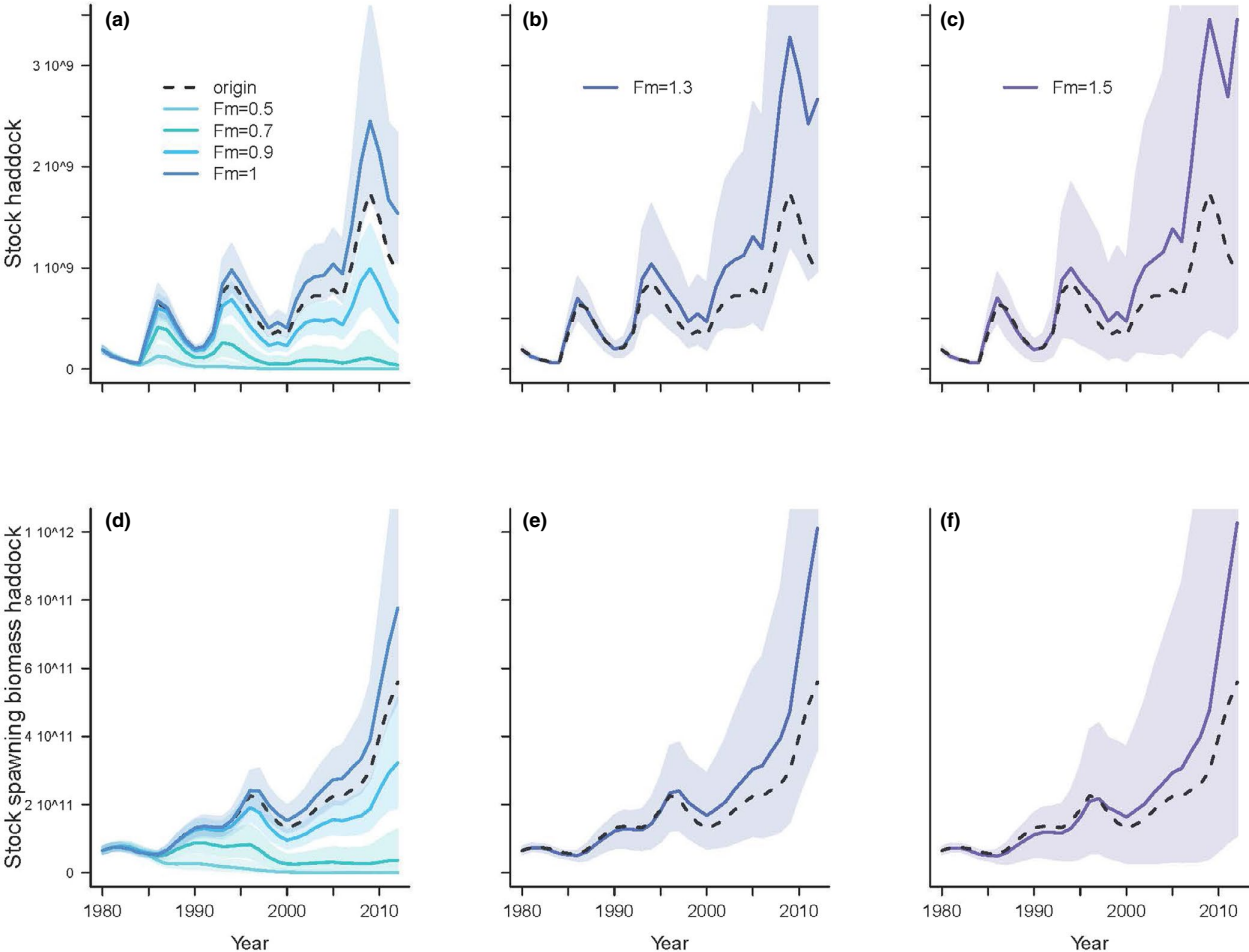
(a–c) Hindcasted abundances (i.e., number of individuals) of haddock stock from the posterior distribution of the parameters (1000 posterior samples) of the haddock life cycle model with harvest intensity (*Fm*) of cod ranging from 0.5 to 1.5 from 1980 to 2012 (i.e., 33 years). (d–f) Hindcasted abundances of stock spawning biomass of haddock (in metric ton) from the posterior distribution of the parameters (1000 posterior samples) of the haddock life cycle model with harvest intensity (*Fm*) of cod ranging from 0.5 to 1.5. The plain line corresponds to the mean hindcasted haddock stock (a–c) or haddock stock spawning biomass (d–f). The shaded areas correspond to the 95% credibility intervals of the hindcasted haddock stock (a–c) or haddock stock spawning biomass (d–f). The dotted line corresponds to the estimated haddock stock (a–c) or haddock stock spawning biomass (d–f) from the haddock life cycle model (Eq. 1 to Eq. 12)

**FIGURE 10:**
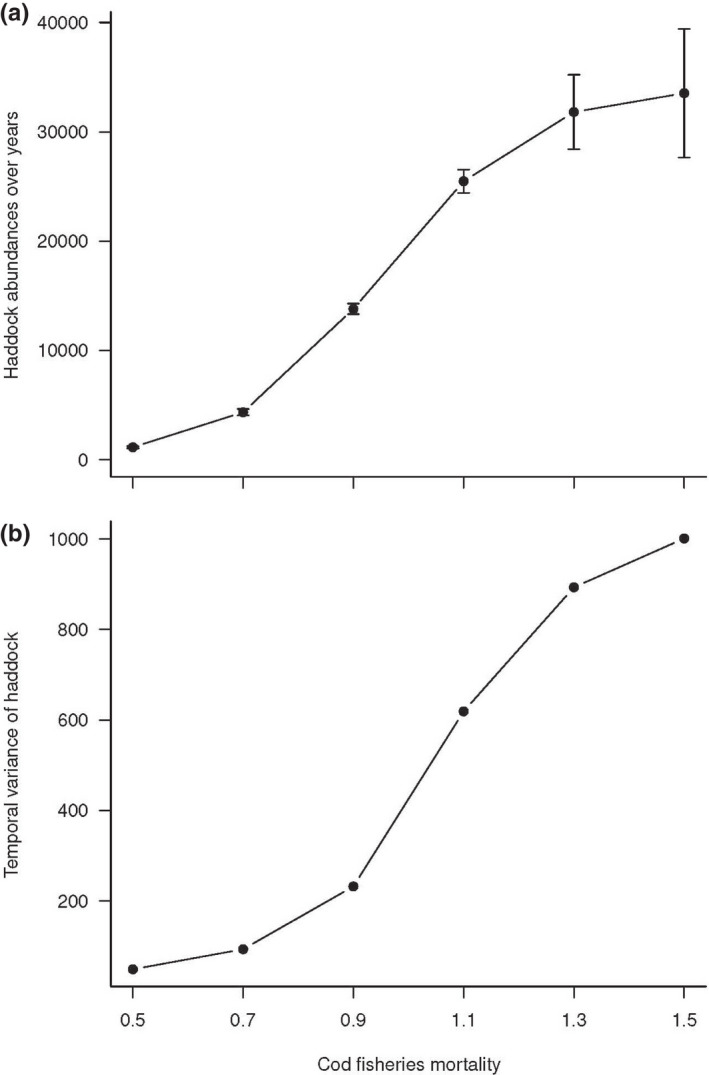
(a) Hindcasted abundances of haddock (numbers of individuals) with increasing fisheries mortality of cod from the posterior distribution of the parameters of the haddock life cycle model. Vertical bars correspond to the 95% confidence intervals estimated as the sum of the squares of the estimates of abundances. (b) Variability of hindcasted abundances of haddock over the time of study with increasing harvest intensity of cod from the posterior distribution of the parameters of the haddock life cycle model

For cod harvest intensity higher than 1.3, increasing of the abundances of cod were dampened (Figure 8). Increasing cod harvest intensity increased the abundances of haddock (Figure 9a–c) and the stock spawning biomass (Figure 9d–f) from 1980 to 2012. The stock spawning biomass of haddock increased notably when the cod harvest intensity increased from 0.5 to 1.3. In the same way, as for the abundance of haddock, the variability of the haddock population increased when the cod harvest intensity increased (Figure 10a). The increase in the haddock population was higher when the cod harvest intensity increased from 0.5 to 1.3 than when the cod harvest intensity was higher than 1.3. The increase in the variability of the abundances of cod was slight when the cod harvest intensity was higher than 1.3 (Figure 10b).

## 4 DISCUSSION

In this study, the novelty holds that I developed a hindcasting (i.e., predicting based on past conditions) approach for two interacting species by accounting thoroughly for complex inter‐stage interactions of the two species, climate variability, and observation errors. I explicitly show that harvest and climate change effects on the dynamics of exploited populations can be transferred through the ecosystem through trophic interactions. It may result in variation of abundances of different species, which can alter the community composition and the ecosystem's functioning (Botsford et al., 1997). This study proposes to lead the way forward for implementing an ecosystem approach to managing exploited populations by showing that it is possible to capture the variation of the population dynamics of interacting species based on empirically estimated interaction parameters from semi‐integrated life cycle state‐space models. I first developed life cycle models for the population dynamics of Atlantic cod (*G. morhua*) and haddock (*M. aeglefinus*) that included information from scientific surveys and commercial harvest. The haddock model extends and corrects the model of Patin et al. (2015), which had only a part of the potential predation by not including the older cod and the non‐spawning individuals. Here, I used the cod biomass of age 3–12, and accounted for all potential predation by cod. Furthermore, as an adjustment of model complexity, I reduced the model by excluding capelin from the model and removing the residual variability in fishing mortality independent of year and age, reflected by *τ_W_*. I fixed the age‐specific mortality for cod older than 4 years. The results show that I obtained more apparent quantification of cod predation on young haddock as age‐0 and age‐1 are positively affected by predation and age‐3 negatively affected by predation (Figure 5). Considering trade‐off in model complexity facilitated the estimation of the state‐space model's parameters and rendered more parsimonious the implementation of the hindcasting approach. To go further, I integrated the use of two single age‐structured life cycle population models, one for the haddock as described in the section *process age*‐*specific abundance model for haddock* and *observation model* (Eq. 1 to Eq. 12) and one for cod as described in section *process age*‐*specific abundance model for cod* (Eq. 13 to Eq. 21). The integration of the two models enabled the analyses of the interactions among life stages of haddock and cod. It quantified the range of potential impact of different harvest intensities on cod in terms of population changes of haddock. The hindcasting approach allowed me to run different harvesting scenarios and track population‐level responses of both species by warding off shortcomings of model forecasting (i.e., future projections), that can lead to an inaccurate accounting of uncertainties and overconfidence in model projection (Brander et al., 2013).

The mortality of haddock was highly variable through age and time. Mortality was the highest at age‐0 and decreased through age. High mortality variability through time might be attributed to the fact that egg production and 0‐group survival are highly stochastic processes, evidenced by the large variance of the process errors (Hutchings & Myers, 1993). Density‐dependence in the juvenile stage was found in age‐0. Indeed, increasing densities of haddock increased the juvenile age‐class mortality (age‐0), suggesting that density‐dependence was compensatory (i.e., increase in adult haddock population decrease the juvenile age‐class size). Density‐dependence seems to decrease when age increase, which can have a dampening effect on cohort fluctuations. These results are in line with the fact that density‐dependence is often assumed to take place during the early life stage in marine fishes (Beverton & Holt, 1957). Similar results were found for cod, for which the population dynamics present compensatory dependence effects, which are dampened through increasing ages (Langangen, Stige, Yaragina, Vikebø, et al., 2014; Ohlberger et al., 2014). Most fish population abilities to overcome disturbances are related to strong‐density‐dependence in early stages, which is issued from variation in mortality (i.e., survival) (Shepherd & Cushing, 1980). This variation in mortality could be due to different factors such as lack of food, predation, and environmental variation (Myers & Cadigan, 1993). Thus, emphasizing the fact that density‐dependent recruitment is essential for the overall population growth and implies interaction among trophic levels simultaneously.

The variation of haddock mortality through age could also be attributed to predation by cod, which is not constant at a different age. Age‐0 and age‐1 of haddock are significantly affected by predation of cod, whereas age‐2 of haddock is not affected by predation of cod. This might explain the fact that the mortality of age‐2 was not so much variable through time. Mortality of age‐3 was negatively affected by predation of cod, which means that the biomass of cod affected the survival of age‐3 haddock positively. This result suggests that cod might prefer other fishes of the same size as age‐3 haddock. Moreover, increasing cod inter‐cohort interactions (i.e., cannibalism) is associated with an increase in haddock age‐3, suggesting that haddock age‐3 is not part of the diet of cod and might reflect an indirect effect of competition (i.e., similar prey). Indeed, the coexistence of competitors can be affected by temporal variation in resources, especially if the time scale of resource population dynamics is fast relative to the time scale of environmental variation (Abrams, 1984). Predation rates on fish larvae are difficult to measure in the field due to a large variety of potential predators, the rapid digestion of fish larvae by predators, and sampling difficulties relating to the patchy distribution of predators (Anderson, 1988). Nevertheless, some studies about the feeding ecology of cod show that cod eats small haddock fishes in the Barents Sea (Yaragina et al., 2011). The nature of the interactions between cod and haddock shifts from predator‐prey to competition around age‐2 to ‐3. The change observed from predator‐prey to competition at age 2–3 could be due to an ontogenetic shift. Indeed, the haddock and cod habitats at age 2–4 are less overlapping than at earlier age (Olsen et al., 2010). The life cycle space‐state model enabled us to quantify the effect of predation of cod on the juveniles of haddock and identify a shift in the type of ecological interactions. Such change in interspecific interaction is rarely explicitly shown and quantified in natural populations, although theoretically described (Holt, 1977; Polis et al., 1989).

The increasing temperature positively affected the number of juveniles of haddock, which is in accordance with other studies suggesting that growth and survival of early life stages of haddock depend on temperature (Langangen, Stige, Yaragina, Ottersen, et al., 2014; Stige et al., 2010). Similar results have also been shown for Northeast Atlantic cod (Ohlberger et al., 2014). Temperature variation can also affect the strength of the interaction between cod and haddock. An increase in cod abundance is associated with increased temperature, negatively affecting the haddock population abundance (Durant et al., 2020). However, the effect of temperature is less critical at age‐0 than for the other juvenile stages. This result suggests that different ecological processes, as the timing between the fish spawning and the onset of annual primary production, might influence the survival of age‐0 (Cushing, 1975). Then, the temperature effect decreased by increasing age, suggesting that older individuals are not affected by increasing temperature. Indeed, the temperature can weakly affect average growth (van Denderen et al., 2020). This result is essential because harvest and climate impose a strong differential selection on natural populations and generate phenotypic changes (Pauly & Cheung, 2018). It emphasizes the importance of the older age classes to keep a stock of marine fish in a good state, robust to climate change (Anderson et al., 2008) and to maintain ecological interactions functioning (Dell et al., 2014).

Increasing cod harvest intensity (i.e., decreasing the abundance of cod) has increased the overall abundance of haddock. Thus, the hindcasting results suggest compensatory dynamics of both species as the decrease in one species is compensated by the increase in the other. However, as cod has a very generalist diet, a decrease of haddock might not be associated with a rise in cod (Bogstad et al., 2000). As observed in George Bank, haddock's egg predation causes alternate stable population levels in herring abundances (Richardson et al., 2011). The underlying mechanism of compensatory dynamics is assumed to be due to competition among species (Gonzalez & Loreau, 2009) and/or the compensation of decline between species in response to environmental change (Vasseur & Gaedke, 2007).

In this study, I consider the one‐way interaction of cod on juvenile mortality of haddock solely. The interaction in the other way, the effect of the haddock on the juveniles of cod, is not included because the effect of haddock on juveniles of cod is minimal; the haddock eat 0.4% of cod in the Barents Sea (ICES, 2008). Therefore, it seems much less important than the effect of cod on juvenile mortality of haddock and the effect of intraspecific cohort interaction for cod (i.e., cannibalism) which are explicitly included and quantified in this study. Moreover, the time series of cod and haddock are not of the same length. The times series of the cod is of 54 years (from 1959 to 2012), and the one of the haddock is of 33 years (from 1980 to 2012) which can generate some statistical estimation issues either if the haddock biomass is included as covariates in the single cod model or if the two models are merged dynamically. Intending to merge both models dynamically using both time series of different lengths, or keeping only the overlapping years of both species did not provide any realistic results. The fully integrated model might be over‐parameterized, potentially unidentifiable, or even not estimable with the available data (Eberly & Carlin, 2000). Nonetheless, it would be interesting to analyze with this framework a two‐species study system with two‐way interactions with long‐term times series of the same length for the two species and data available for the entire life cycle of both species for more fit in the frame of the theoretical Lokta‐Volterra model (Lotka, 1925; Volterra, 1931). Other species such as long rough dab, saithe, Greenland halibut, capelin, and herring also interact trophically with cod and haddock (ICES, 2008, 2013); haddock is prey for Greenland halibut, long rough dab, and saithe (ICES, 2008). These interactions might be interesting to analyze further in a meta‐analysis.

Overall, the results show that haddock's early stages are density‐dependent, influenced by temperature, and predated by cod. In contrast, haddock adult stages are density‐independent, less influenced by temperature, and affected by the level of intraspecific interaction of cod (i.e., cannibalism). These results suggest that competition might be the primary driver of the overall dynamics of the interaction between both species at adult stages. At early stages, the predation prevails; thus, if the prey species is affected by environmental disturbances, adjusting the harvesting intensity of the early stage predator species might dampen the effects of the environmental disturbances so that both species continue to fluctuate around an equilibrium.

## CONFLICT OF INTEREST

The author has no conflict of interest.

## AUTHOR CONTRIBUTION

**Edwige Bellier:** Conceptualization (lead); Data curation (lead); Formal analysis (lead); Investigation (lead); Methodology (lead); Writing – original draft (lead); Writing – review & editing (lead).

## Supporting information

Supplementary Material

## Data Availability

The data used in this study are publicly available. More explanations are provided in the section “*Data description*” in the Supporting Information. The data can also be available from the corresponding author on reasonable request.
