# Supplementary material for: Mixed interactions among life history stages of two harvested related species
Source: Ecol Evol. 2022 Mar 7;12(3):e8530. doi: 10.1002/ece3.8530 (PMC8901886; doi:10.1002/ece3.8530)
Supplement: Supplementary file 1 — Supplementary Material [file ECE3-12-e8530-s001.pdf]

Supporting Information

Mixed interactions among life history stages of two harvested related species

Edwige Bellier

The Arctic University of Norway, Department of Arctic and Marine Biology, 9037 Tromsø, Norway.

Corresponding author: Edwige Bellier, present address: Warnell School of Forestry and Natural Resources, University of Georgia, 180 E. Green Street, Athens, GA 30602, USA.

Email: edwbellier@gmail.com ; Phone: (+1) 706 542 9885.

Data description

The model includes information from scientific surveys and commercial harvest since 1980 up until now a day (i.e., from 1980 to 2012, 33 years). Reported landing and abundance estimates for NEA haddock are publicly available in ICES Report (ICES 2013). Data on the 0-group and larvae of NEA haddock as well as the sea temperature in Kola section (i.e., area of spawning and recruitment of NEA cod and haddock) are publicly available in the Survey report from the joint Norwegian/Russian ecosystem survey in the Barents Sea and adjacent waters (Prozorkevich *et al.* 2018). Egg data of NEA haddock are freely accessible from a study that predicts fish recruitment from juvenile abundance and environmental indices (Stige *et al.* 2013). Cod estimates of biomass were obtained from the number of individuals of cod of ages 3-12 estimated from the cod model described in (Ohlberger *et al.* 2014) and the weight of cod at age 3-12 provided in ICES report (ICES 2013). (see Fig. 3 for a schematic representation of the different kind of data). I used the estimates of the cod inter-cohort interaction from the age-structured cod model (Ohlberger *et al.* 2014) to analyze the effect of the cod inter-cohort interactions on the variation of the abundances of haddock.

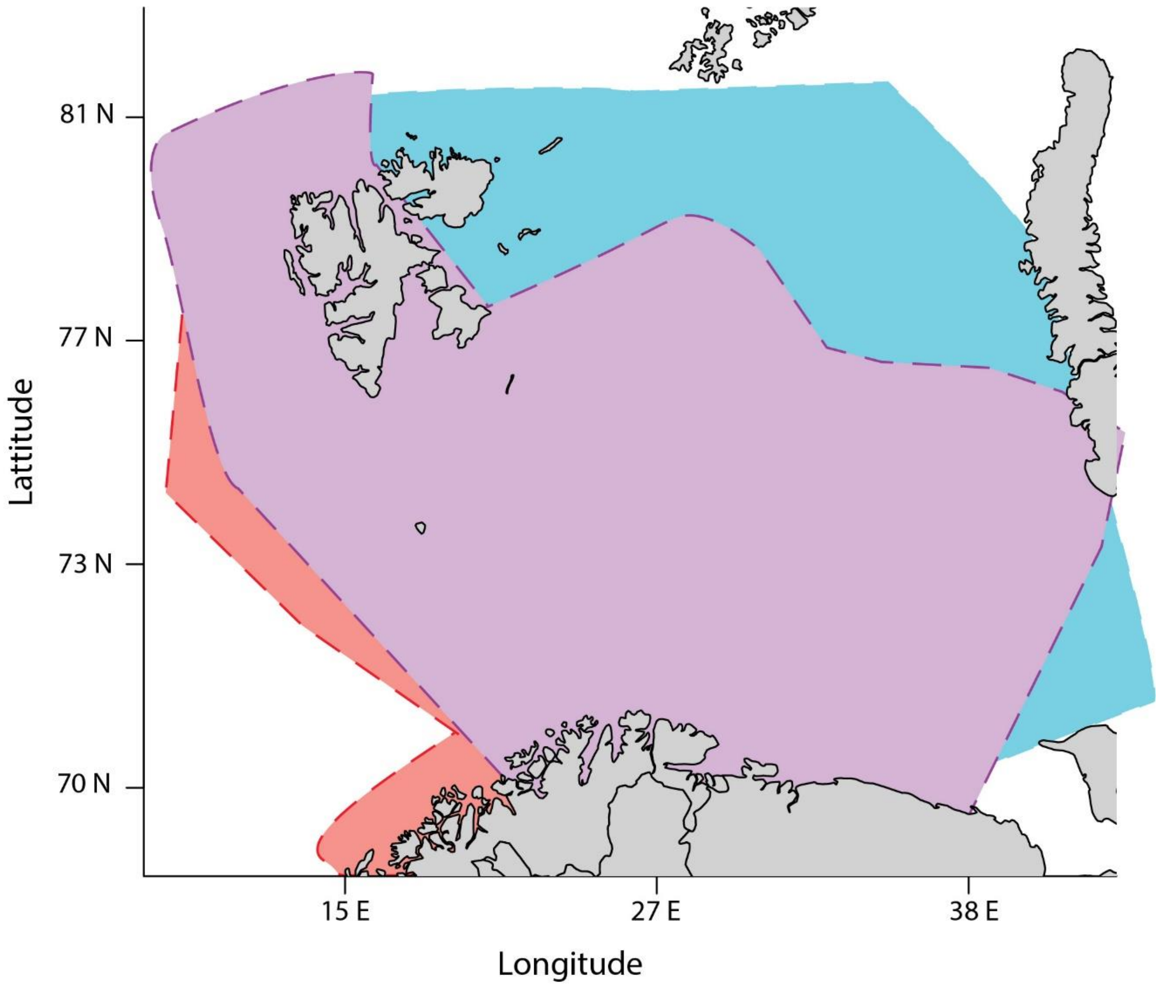

Figure S1. Schematic spatial distribution of haddock (red) and cod (blue) in the Barents Sea. The purple area corresponds of the overlapping distribution of both species. The map has been drawn from Bakketeig *et al.* (2016).

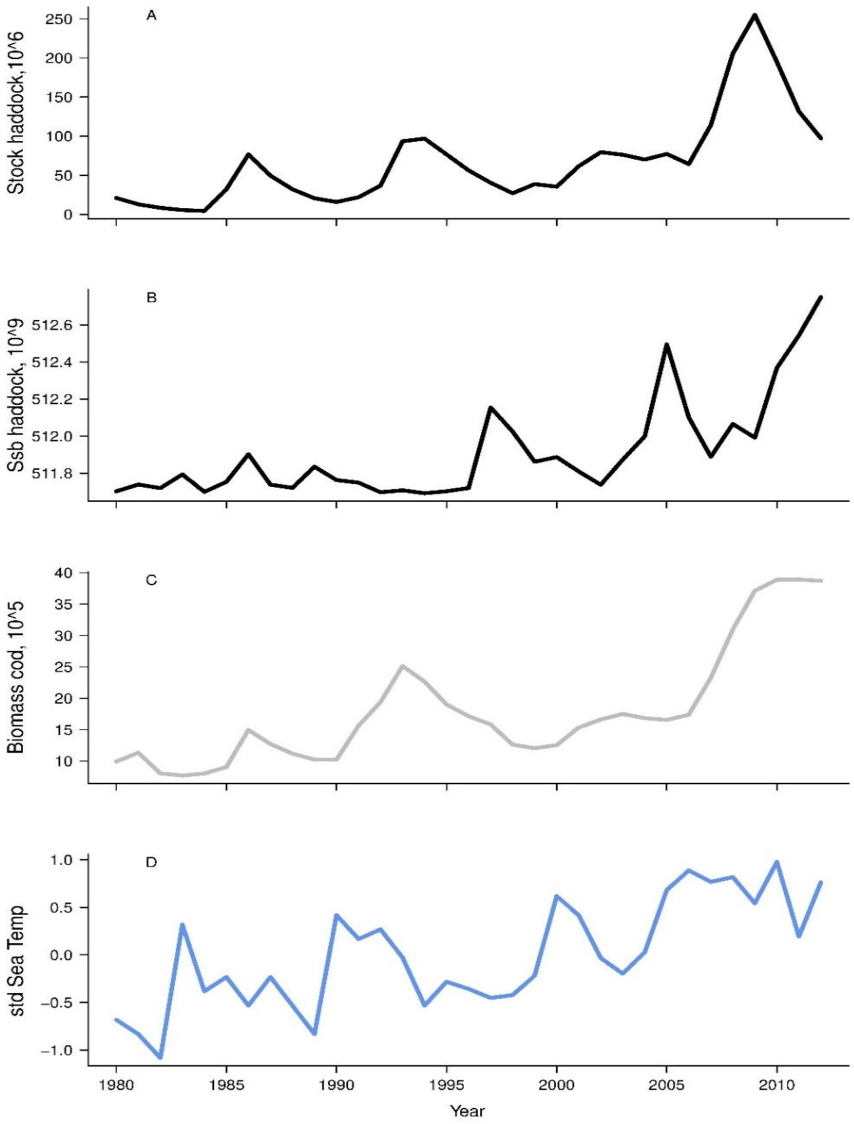

Figure S2. (A) Estimated haddock population (numbers of individuals Age 3-14+) from 1980 to 2012 (i.e., 33 years) as reported in the ICES report (ICES 2013). (B) Estimated stock spawning biomass of haddock (in metric tons) from 1980 to 2012 estimated from ICES report (ICES 2013). (C) Cod biomass (in metric tons) from 1980 to 2012 estimated from Ohlberger *et al.* (2014). (D) Standardized sea temperature at Kola section during winter (December to April) averaged over the water column (0-200 m) from 1980 to 2012 as given in Prozorkevich *et al.* (2018)**.**

Table S1. Connection between the different steps in the quantitative estimation of the species interactions and hindasting framework and the results as described in Fig. 3.

Figures	Analyses	Steps in the framework
**Fig. 5**	Estimation of the predation on haddock juvenile by cod at different life-stages (i.e., age).	**Step 1a** - Quantitative estimates, haddock (eq. 1 to eq. 12) state-space model.
**Fig. 6**	Effect of temperature on abundances of haddock at different life-stages (i.e., age).	**Step 1a** - Quantitative estimates, haddock (eq. 1 to eq. 12) state-space model.
**Fig. 7**	Effect of inter-cohort interaction of cod on abundances of haddock at different life-stages (i.e., age).	**Step 1a and 1b** - Quantitative estimates, haddock (eq. 1 to eq. 12) and cod (eq. 13 to eq. 21) state-space model.
**Fig. 8**	Simulated abundances of cod from the posterior distribution of the parameters of the cod life-cycle state-space model with different harvest intensity of cod.	**Step 2a** - Hindcasting procedure as described in Fig. 4.
**Fig. 9**	Simulated abundances of haddock stock over time from the posterior distribution of the parameters of the haddock life-cycle state-space model including the interaction with cod with different harvest intensity of cod.	**Step 2b** - Hindcasting procedure as described in Fig. 4.
**Fig. 10**	Simulated abundances of haddock from the posterior distribution of the parameters of the haddock life-cycle state-space model with different harvest intensity of cod.	**Step 2b** - Hindcasting procedure as described in Fig. 4.

Table S2. Description of the prior distribution for the main parameters of the haddock life cycle mode (Eq. 1 to Eq. 12).

Variable	Definition	Prior distribution	Life-stage/age-class
D_a	Age-specific density-dependence	log-N(log(10^{-10},0.04)	Age0 - 3
g_a	Age-specific predation	N(0,10^{10})	Age 0- 3
T_φ	Temperature dependent effect	N(0,0.01)	Age 0
m_0	Natural mortality	log-N(log(2.05), 0.1)	Age 0
m_1	--	log-N(log(1.65), 0.1)	Age 1
m_2	--	log-N(log(0.4), 0.1)	Age 2
m_3	--	log-N(log(0.25), 0.1)	Age 3
m_4	--	log-N(log(0.2), 0.1)	Age 4
σ_φ	Process error variance number of juveniles	U(0,5)	Age 0
f_a	Age specific fishing mortality	Log-N(log(0,0.04))	Age 3-14
σ_w	Variance harvest mortality random effect	U(0,0.01)	Age 3-14
σ_F	Year effect on harvest mortality	U(0,0.01)	Age 3-14
σ_{La}	Year effect on harvest mortality	U(0,5)	Age 3-14
σ_{I_0}	Age specific abundance indices error	U(0,5)	Age 3-14
σ_{Ia}	Age specific abundance indices error	U(0,5)	Age 0
σ_{La}	Age specific abundance catches error	U(0,5)	Age 1-5
q_z	Catchabilities survey	log N(0,0.0002)	Age 3-14
q_a	Catchabilities survey	Log N (0,0.002)	Age 0

Table S3. Estimated main parameters of the haddock life cycle mode with 95 % credibility intervals (Eq. 1 to Eq. 12).

Variable	Median	95 % Credible	Interval	Life-stage/age-class
Age specific density-dependence				
D_0	1.212e-11	4.019e-15	9.523e-11	0-group
D_1	1.852e-12	1.128e-15	7.591e-11	Age 1
D_2	2.637e-12	1.479e-15	1.213e-10	Age 2
D_3	3.286e-12	1.440e-15	1.505e-10	Age 3
Age-specific predation				
g_0	2.651e-07	1.361e-07	4.302e-07	0-group
g_1	1.681e-07	4.123e-08	3.221e-07	Age 1
g_2	4.196e-08	-4.040e-07	4.083e-07	Age 2
g_3	-9.426e07	-2.470e-06	-2.125e-09	Age 2
Correction for unbiased relationship for age 0-3 mortalities				
δ_0	-0.529	-0.863	-0.253	0-group
δ_1	-0.324	-0.628	-0.0748	Age 1
δ_2	-0.073	-0.815	0.668	Age 2
δ_3	1.478	0.004	3.240	Age 2
Correction for unbiased relationship for stock recruitment				
δ_{Tfec}	-0.104	-0.010	-0.261	0-group
Mortalities rates				
m_0	1.456	0.958	1.975	0-group
m_1	1.146	0.708	1.695	Age 1
m_2	0.336	0.181	0.584	Age 2
m_3	0.188	0.107	0.324	Age 3
Temperature effect on age-0				
T_φ	0.787	0.258	1.323	0-group
Process error abundance age-0				
σ_φ	0.743	0.559	1.013	0-group

Table S4. Estimated fishing mortality of the haddock with 95 % credibility intervals (Eq. 9).

Variable	Median	95 % Credible Interval		Life-stage/age-class
Fishing mortality in first year for age 3-14+				
f_3	1.080	1.025	1.165	Age 3
f_4	1.210	1.136	1.309	Age 4
f_5	1.414	1.304	1.551	Age 5
f_6	1.588	1.439	1.771	Age 6
f_7	1.603	1.434	1.810	Age 7
f_8	1.541	1.374	1.742	Age 8
f_9	1.372	1.232	1.534	Age 9
f_{10}	1.426	1.238	1.628	Age 10
f_{11}	1.310	1.151	1.489	Age 11
f_{12}	1.493	1.195	1.839	Age 12
f_{13}	1.314	1.103	1.576	Age .13
f_{14}	1.265	1.030	1.873	Age 14

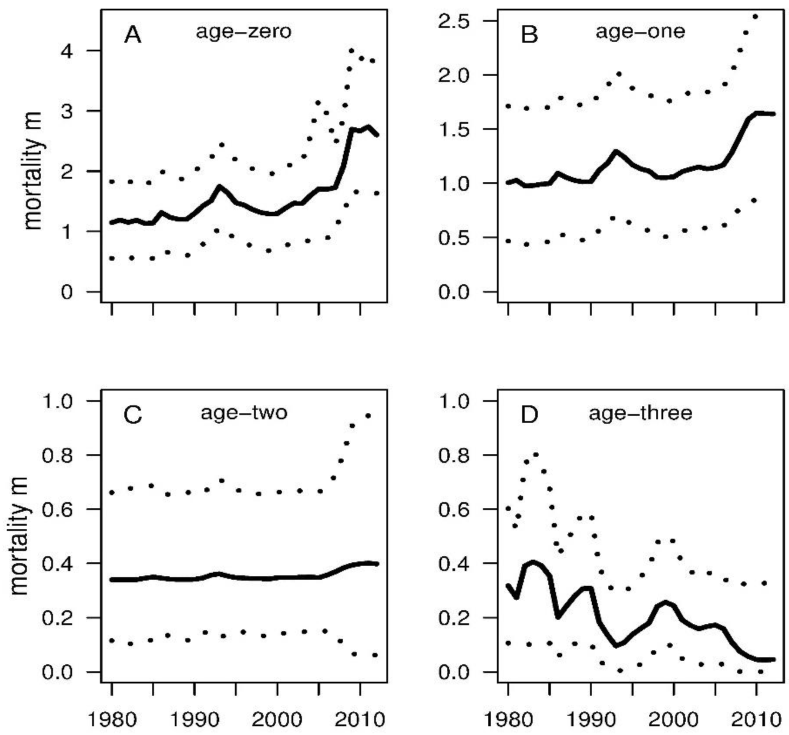

Figure S3. Estimated mortality of haddock from Eq. 7 as a function of year. (A) Estimated mortality of haddock at age-0 as function of years. (B) Estimated mortality of haddock at age-1 as function of years. (C) Estimated mortality of haddock at age-2 as function of years. (D) Estimated mortality of haddock at age-3 as function of years. The black plain line corresponds to the estimated mortality. The dotted black line corresponds to the 95 % credibility intervals.

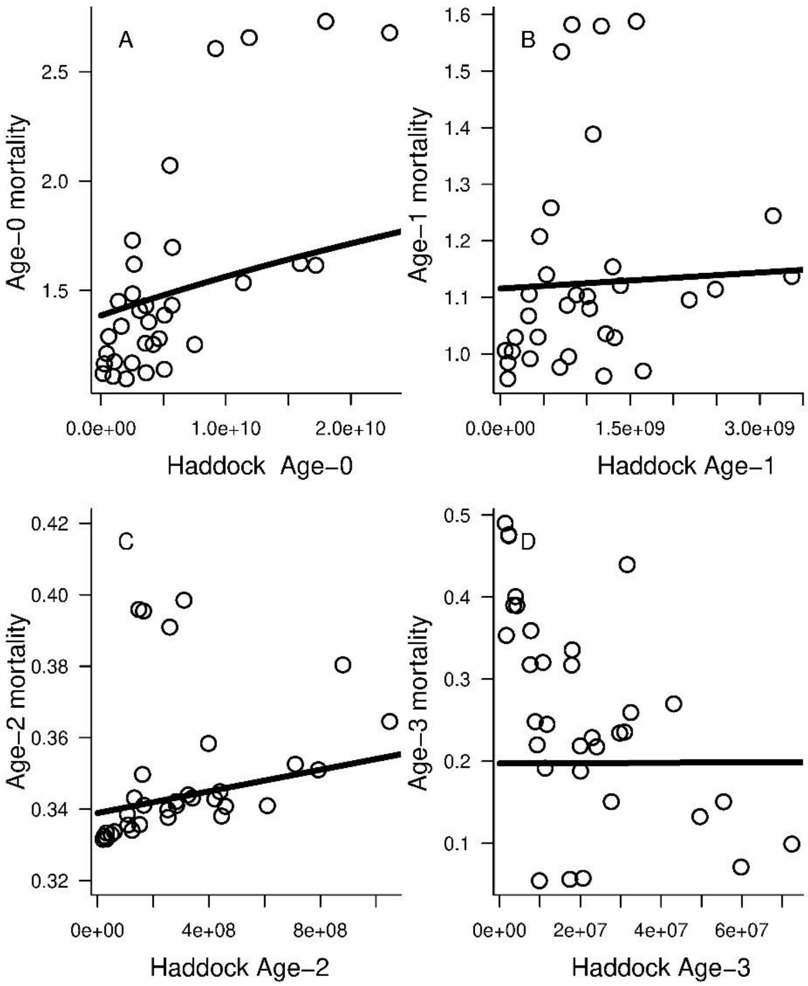

Figure S4. (A-D) Mortality at from age-0 to age-3 as function of the estimated number of individuals of haddock. The black plain line corresponds to the contribution of density dependence to the mortality (Eq. 7).

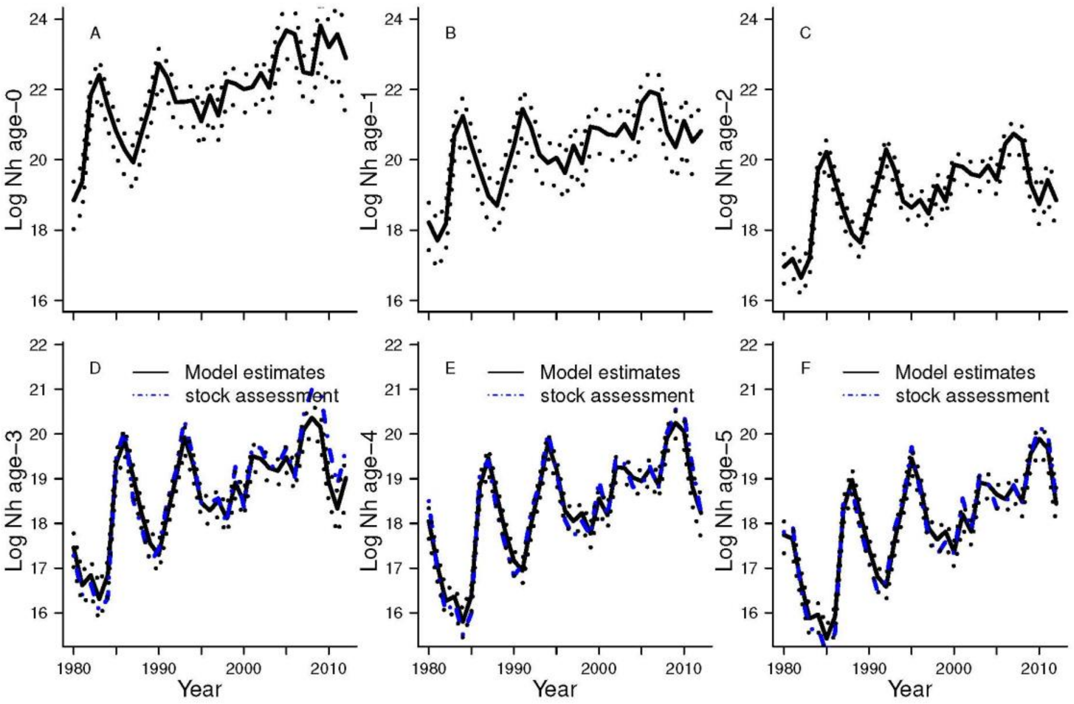

Figure S5. Estimated log-number of individuals of haddock (Eq. 2) from age-0 to age-5 (A to F) from 1980 to 2012. The black line corresponds to the mean estimates, the dotted line to the 95 % credibility intervals. The blue dotted line corresponds to the estimates of the stock assessment provided in ICES report (ICES 2013).

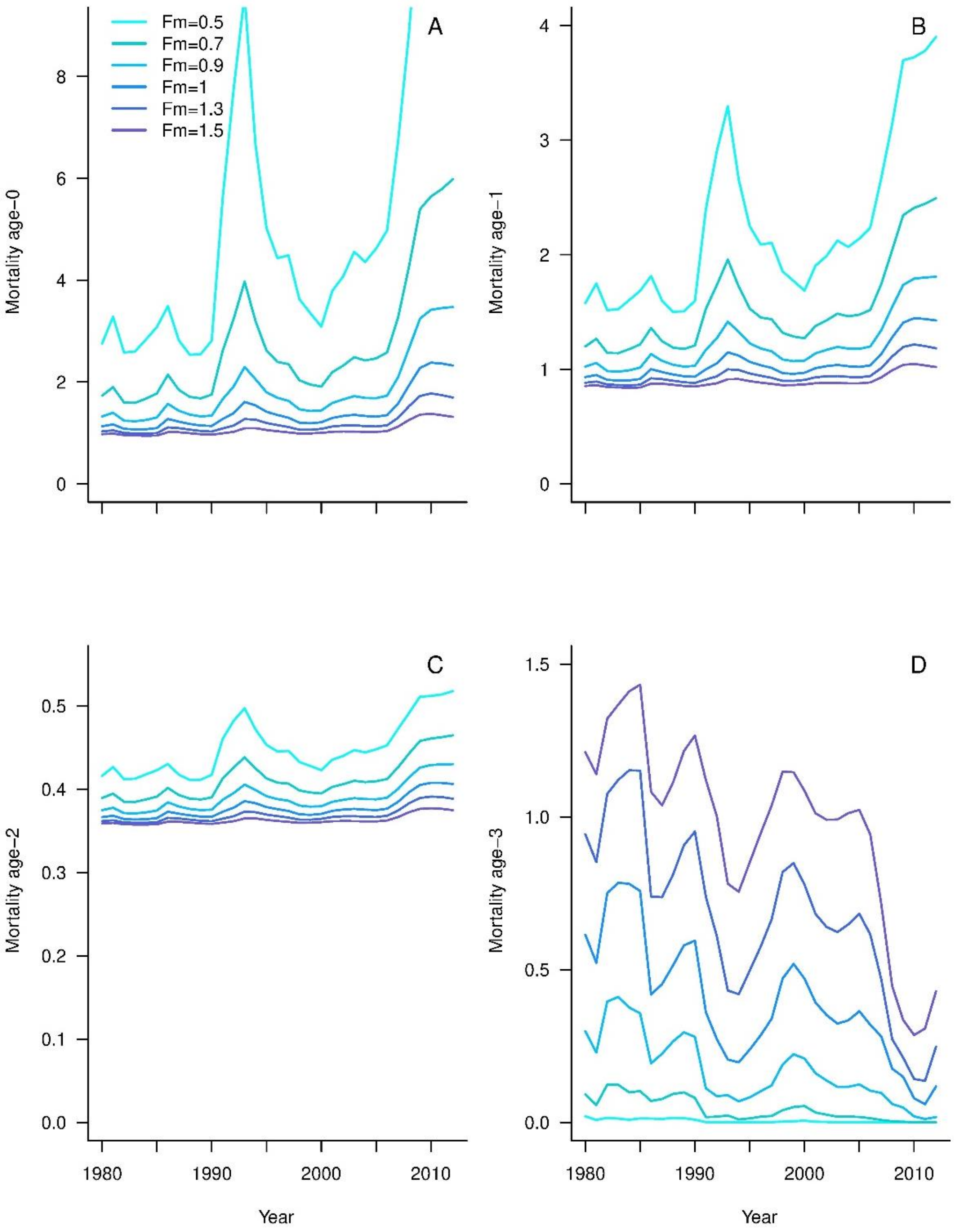

Figure S6. (A-D) Simulated (i.e., hindcasting from 1000 posterior samples) mortalities of haddock for age-0 to age-3 from the life-cycle cod model as described in Eq. 7 including the interactions with cod with different harvest intensity (*Fm*) ranging from 0.5 to 1.5 from 1980 to 2012.

Diagnostic plots for haddock model

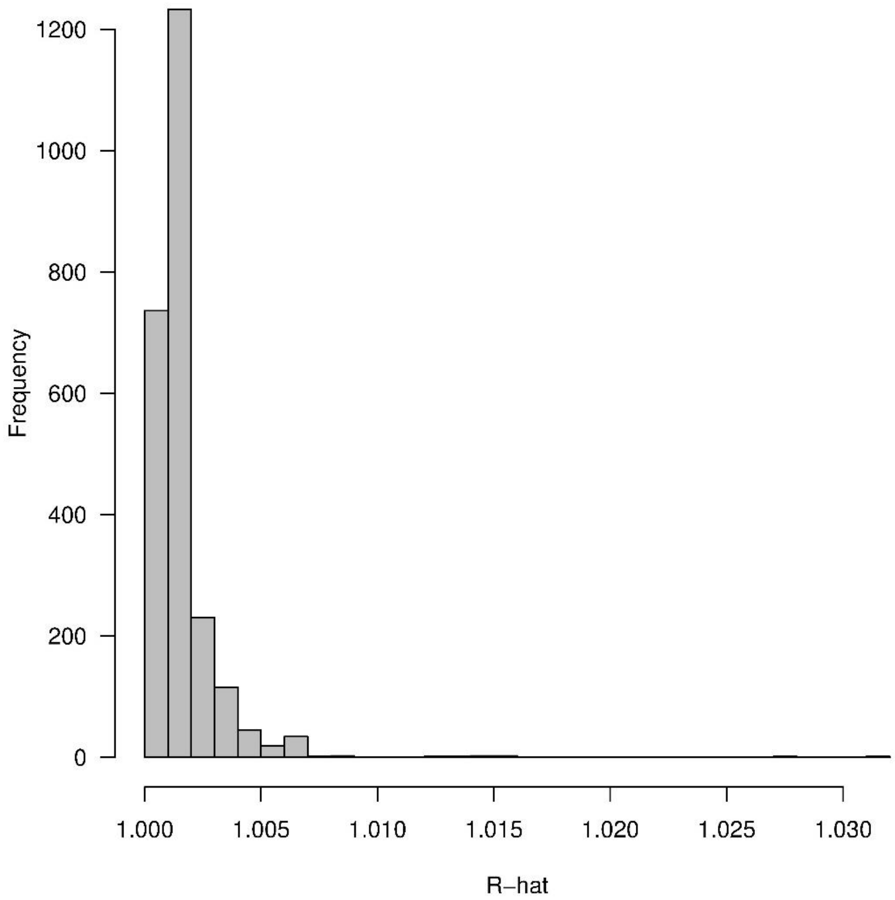

Figure S7. Histogram of all R-hat statistics values representing the Brooks-Gelman diagnostic (Gelman *et al.* 2004) for all the parameters of the haddock model. All the R-hat values had lower values than 1.1.

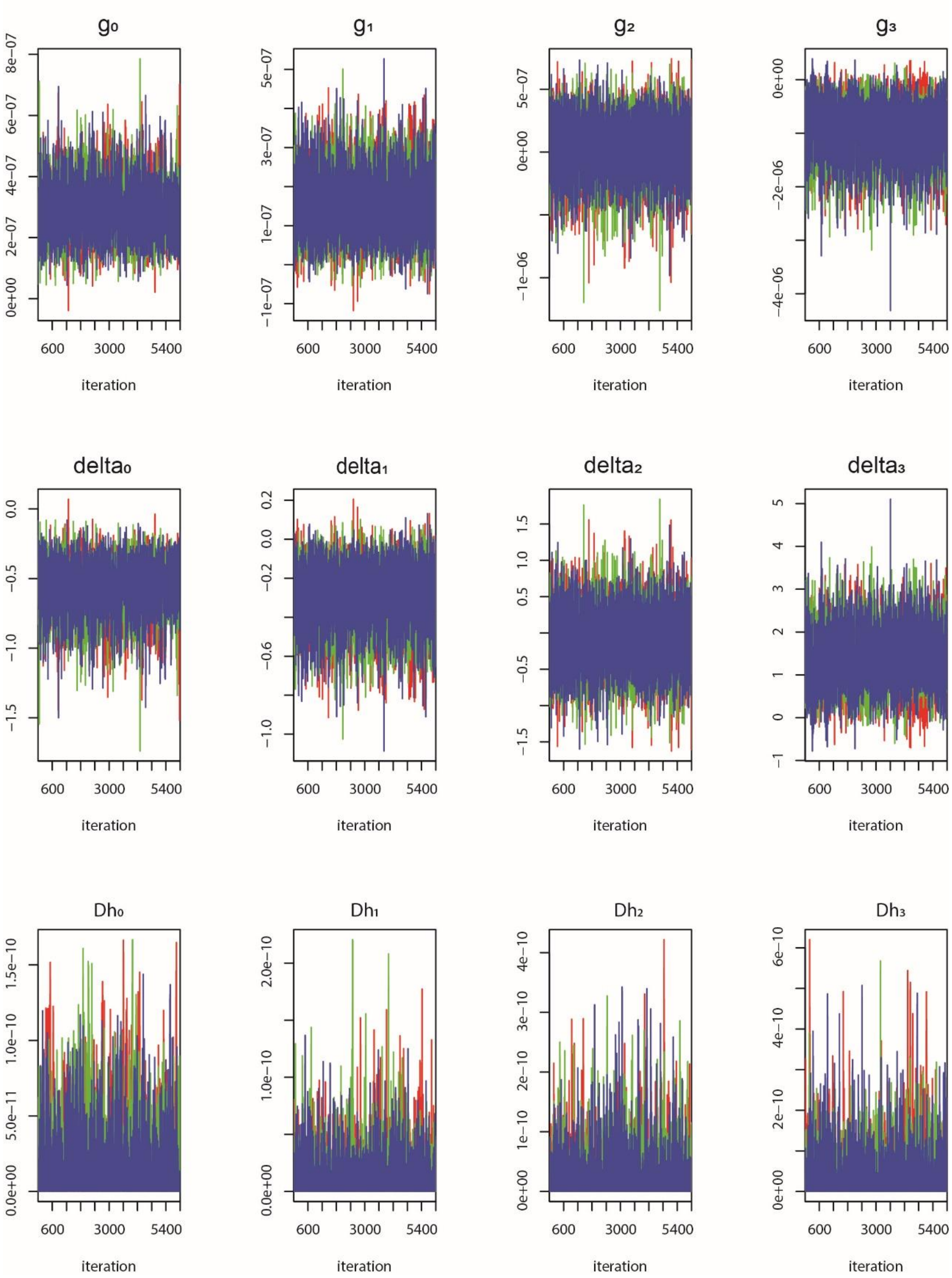

Figure S8. Sampled values as function of iterations for the parameters related to the estimates of haddock's mortality at different ages (age-0 to age-3) (Eq. 6) for the 3 chains.

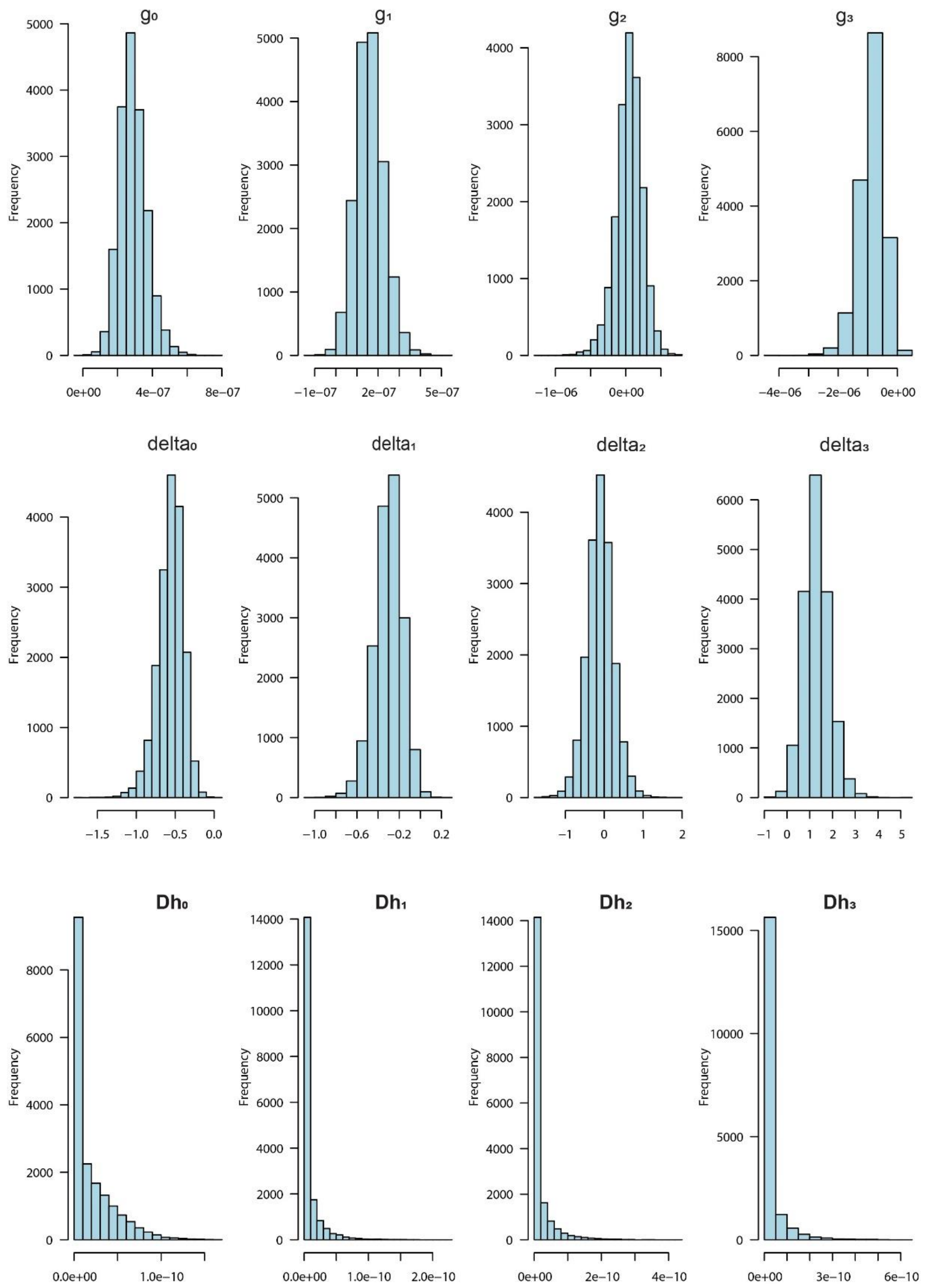

Figure S9. Posterior distribution of the parameters related to the estimates of haddock's mortality at different ages (age-0 to age-3) (Eq. 6) for the 3 chains.

Diagnostic plots for cod model

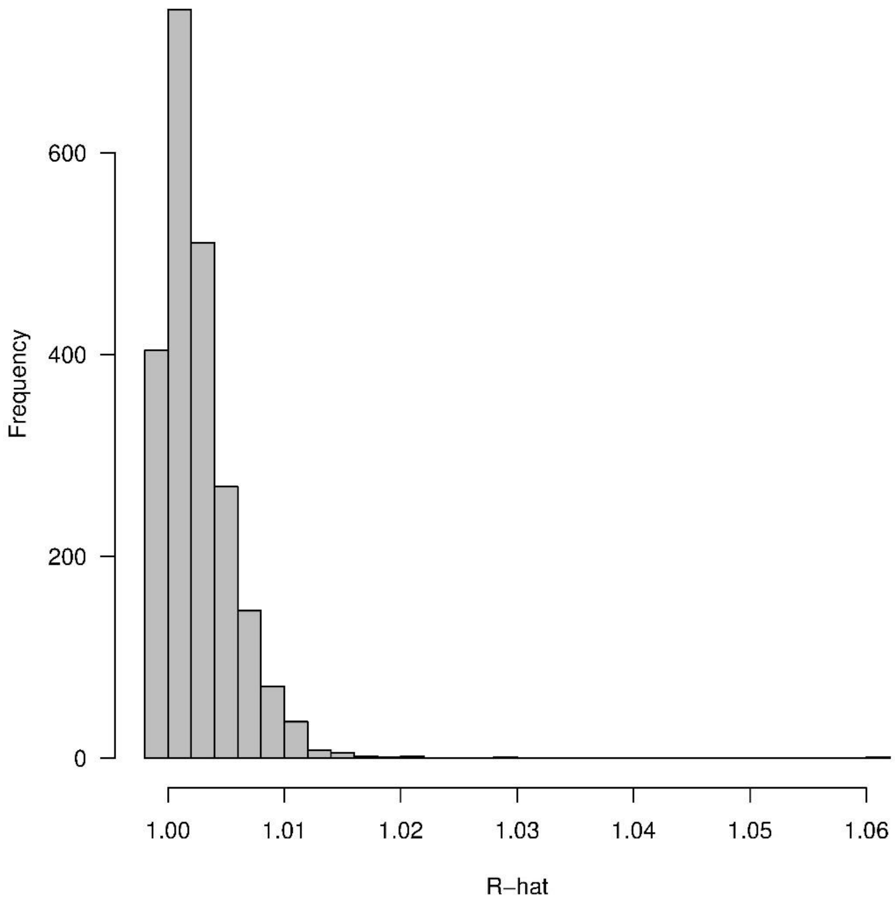

Figure S10. Histogram of all R-hat statistics values representing the Brooks-Gelman diagnostic (Gelman *et al.* 2004) for all the parameters of the cod model. All the R-hat values had lower values than 1.1.

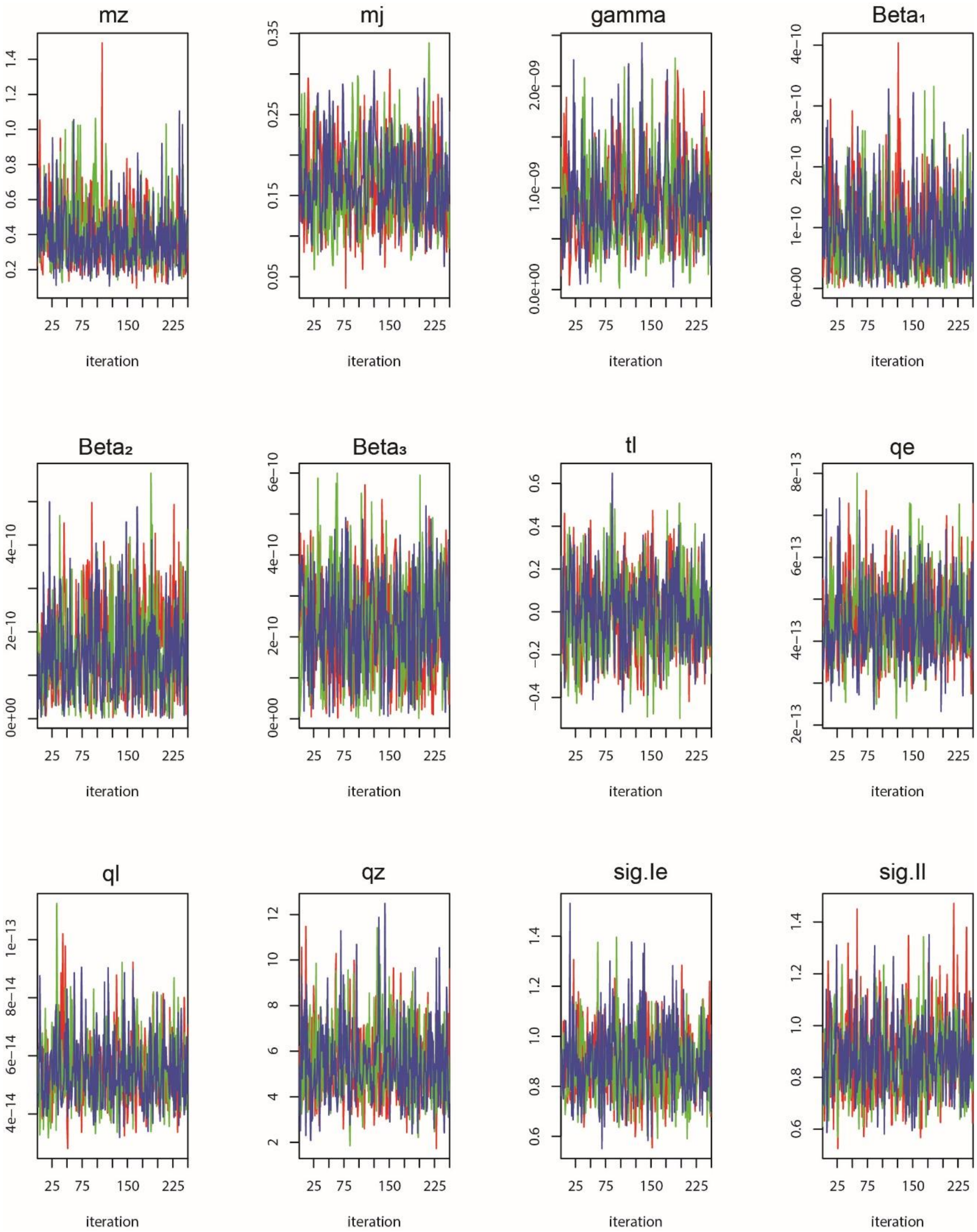

Figure S11. Sampled values as function of iterations for the parameters related to the estimates of cod's survival and fishing mortality (Eq.15 to Eq. 21 in the main text) for the 3 chains.

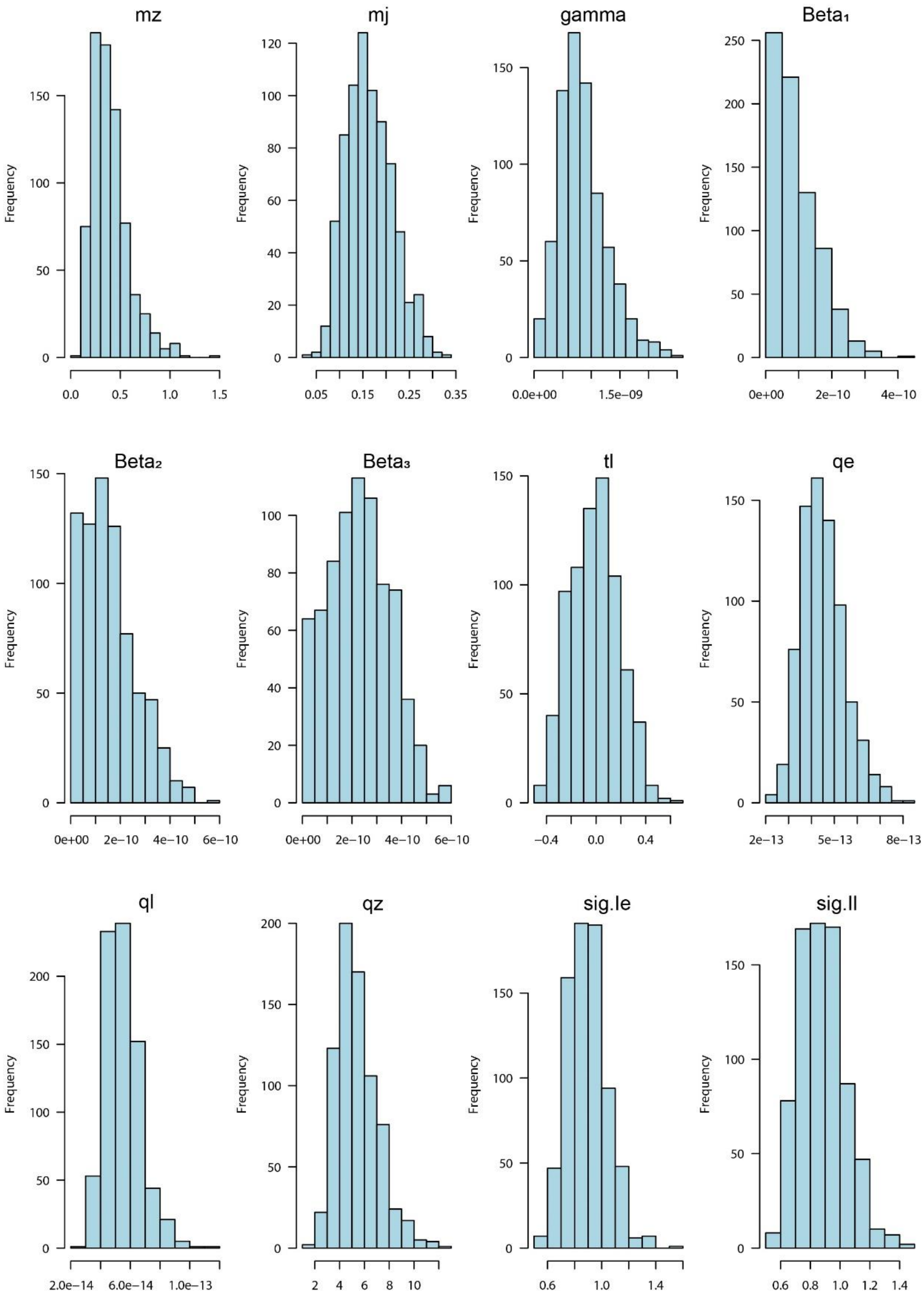

Figure S12. Posterior distribution of the parameters related to the estimates of cod's survival and fishing mortality (Eq.15 to Eq. 21 in the main text).

REFERENCES

Bakketeig, I.E., Hauge, M., Kvamme, C., Sunnset, B.H. & Toft, K.Ø. (2016). Havforskningsrapporten 2016. In: *Fisken Havet*, pp. 1-203.

Gelman, A., Carlin, J.B., Stern, H.S. & Rubin, D.B. (2004). *Bayesian data analysis* 2nd edn. Chapman and Hall.

ICES (2013). Report of the Arctic Fisheries working group (AFWG). In: *ACOM:05* (ed. 2013, IC). ICES Headquarters Copenhaguen, Danemark.

Ohlberger, J., Rogers, L.A. & Stenseth, N.C. (2014). Stochasticity and determinism: How density-independent and density-dependent processes affect population variability. *Plos One*, 9, e98940.

Prozorkevich, D., Johansen, G.O. & van der Meeren, G.I. (2018). Survey report from the joint Norwegian/Russian ecosystem survey in the Barents Sea and adjacent waters, August-October 2017. In: *IMR/PINRO Joint Rep. Ser.*, p. 98.

Stige, L.C., Hunsicker, M.E., Bailey, K.M., Yaragina, N.A. & Hunt, G.J. (2013). Predicting fish recruitment from juvenile abundance and environmental indices. *Marine Ecology Progress Series*, 480, 245-261.
